# Qualia: The Geometry of Integrated Information

**DOI:** 10.1371/journal.pcbi.1000462

**Published:** 2009-08-14

**Authors:** David Balduzzi, Giulio Tononi

**Affiliations:** Department of Psychiatry, University of Wisconsin, Madison, Wisconsin, United States of America; University College London, United Kingdom

## Abstract

According to the integrated information theory, the quantity of consciousness is the amount of integrated information generated by a complex of elements, and the quality of experience is specified by the informational relationships it generates. This paper outlines a framework for characterizing the informational relationships generated by such systems. Qualia space (Q) is a space having an axis for each possible state (activity pattern) of a complex. Within Q, each submechanism specifies a point corresponding to a repertoire of system states. Arrows between repertoires in Q define informational relationships. Together, these arrows specify a quale—a shape that completely and univocally characterizes the quality of a conscious experience. Φ— the height of this shape—is the quantity of consciousness associated with the experience. Entanglement measures how irreducible informational relationships are to their component relationships, specifying concepts and modes. Several corollaries follow from these premises. The quale is determined by both the mechanism and state of the system. Thus, two different systems having identical activity patterns may generate different qualia. Conversely, the same quale may be generated by two systems that differ in both activity and connectivity. Both active and inactive elements specify a quale, but elements that are inactivated do not. Also, the activation of an element affects experience by changing the shape of the quale. The subdivision of experience into modalities and submodalities corresponds to subshapes in Q. In principle, different aspects of experience may be classified as different shapes in Q, and the similarity between experiences reduces to similarities between shapes. Finally, specific qualities, such as the “redness” of red, while generated by a local mechanism, cannot be reduced to it, but require considering the entire quale. Ultimately, the present framework may offer a principled way for translating qualitative properties of experience into mathematics.

## Author Summary

In prior work, we suggested that consciousness has to do with integrated information, which was defined as the amount of information generated by a system in a given state, above and beyond the information generated independently by its parts. In the present paper, we move from computing the *quantity* of integrated information to describing the structure or *quality* of the integrated information unfolded by interactions in the system. We take a geometric approach, introducing the notion of a quale as a shape that embodies the entire set of informational relationships generated by interactions in the system. The paper investigates how features of the quale relate to properties of the underlying system and also to basic features of experience, providing the beginnings of a mathematical dictionary relating neurophysiology to the geometry of the quale and the geometry to phenomenology.

## Introduction

Consciousness poses two main problems [1]. First, what are the necessary and sufficient conditions that determine the *quantity* of consciousness generated by a system? Is a system enjoying vivid experiences, is it dimly aware, or is it completely unconscious? We know that the corticothalamic system (or parts of it) generates an incessant stream of experience, which only ceases when we fall into dreamless sleep, or when the cortex is severely damaged. By contrast, the cerebellum - a part of our brain as complicated and even richer in neurons than the cortex – does not seem to generate much experience at all: if the cerebellum has to be removed surgically, consciousness is hardly affected. What is special about the corticothalamic system, then, that is not shared by the cerebellum?

Second, what are the necessary and sufficient conditions that determine the *quality* of consciousness? What makes an experience visual, auditory, or both? What makes a color a color, and red red, and what makes red different from blue, a triangular shape, or a high C on an oboe? Again, empirical evidence indicates that different parts of the cortex influence different qualitative aspects of consciousness. For example, damage to certain parts of the cortex can impair the experience of color, whereas other lesions may prevent you from experiencing visual shapes, and other lesions may abolish auditory, rather than visual perception. Why is this so?

The integrated information theory (IIT) [1] attempts to provide a principled answer to these questions. By starting from phenomenology and making a critical use of thought experiments, the IIT claims that: i) the quantity of consciousness is the amount of integrated information generated by a complex of elements; ii) the quality of consciousness is specified by the set of informational relationships generated among the elements of a complex.

### The quantity of integrated information

Informativeness is a key property of consciousness, as can be realized by considering the photodiode thought experiment [1]. Briefly, you and a photodiode face a blank screen that is alternately on and off. When you look at the screen, you “see” light or dark. The photodiode can also discriminate between the screen being on or off, but presumably it does not consciously see anything. According to the IIT, the key difference between you and the photodiode has to do with how much *information* is generated when the discrimination is made. Information is classically defined as reduction of uncertainty. When the blank screen turns on, the mechanism in the photodiode discriminates between 2 alternatives (the current from the sensor is above rather than below a threshold) and thereby generates log_2_(2) = 1 bit of information. On the other hand, when you “see” light, the mechanisms in your corticothalamic system discriminate among a much large number of alternatives: all other experiences you could possibly have had, but did not have (a dark screen, to be sure, but also a blue screen, a checkerboard screen, any frame from any possible movie, with or without any possible sound, and so on). Thus, you generate a much larger amount of information.

Information is not enough, however, if it is not integrated. Consider an idealized digital camera whose sensor chip is made up of 1 million binary photodiodes. Though such a camera could discriminate among a very large number of states (2^1,000,000^, corresponding to 1,000,000 bits of information), it is hard to imagine that it would be generating vivid experiences. According to the IIT, the key difference between you and the camera has to do with *integrated information*. From the perspective of an external observer, the camera chip has a large repertoire of states. From an intrinsic perspective, however, the sensor chip can be considered as a collection of one million photodiodes with a repertoire of two states each, rather than as a single integrated system with a repertoire of 2^1,000,000^ states. This is because, due to the absence of interactions among the photodiodes within the sensory chip, the state of each element is causally independent of that of the other elements. Indeed, if the sensor chip were literally cut down into individual photodiodes, the performance of the camera would not change at all. By contrast, the repertoire of states available to you cannot be subdivided into the repertoire of states available to independent components. This is evident phenomenologically: when you consciously “see” a certain image, that image is experienced as an integrated whole and cannot be subdivided into component images that are experienced independently, such as the left half of the visual field of view independently of the right half. Underlying this unity is a multitude of causal interactions among the relevant parts of your brain. Indeed, unlike disconnecting the photodiodes in a camera sensor, disconnecting brain regions has profound effects. For example, when the 200 million fibers linking the two cortical hemispheres are cut to alleviate severe seizures, consciousness literally splits in two [2]: the left hemisphere experiences the right half of the visual field, the right hemisphere the left half, and nobody sees the whole picture.

Based on these considerations, the IIT goes on to claim that the quantity of consciousness of a physical system is related to the repertoire of different states (information) that can be discriminated by the system as a whole (integration). A measure of integrated information, called phi (Φ), can be used to quantify the information generated when a system is in a particular state of its repertoire, above and beyond the information generated independently by its parts [1].

### The quality of integrated information

If the amount of integrated information generated by a system can in principle account for changes in the level of consciousness, what is responsible for the quality of each particular experience? For example, one can be aware of pure red on one instance, and of a piercing sound on another instance. In both instances, one is aware with roughly the same intensity – the quantity of consciousness is similar – but the quality of the experience is radically different. What determines that colors look the way they do, and different from the way music sounds? And why do different cortical areas seemingly contribute different qualities to experience? Why does damage to certain parts of the cerebral cortex forever eliminates our ability to experience color (whether perceived, imagined, remembered or dreamt), whereas damage to other parts selectively eliminates our ability to experience visual shapes?

The IIT claims that, just like the quantity of consciousness generated by a complex of elements is determined by the amount of integrated information it generates, the quality of consciousness is determined by the set of informational relationships its mechanisms generate [1]. Consider again the photodiode thought experiment. When the photodiode reacts to light, it can only tell that things are one way rather than another way. On the other hand, when we see “light,” we discriminate against many more states of affairs as a single entity, and thus generate much more integrated information, i.e. consciousness. But what makes “light” light, and not some other conscious experience? The key is to realize that the many discriminations we can do, and the photodiode cannot, do not merely distinguish some particular state against an undifferentiated bunch of equivalent alternatives, but rather discriminate that state, in a specific way, against each and every alternative.

Consider a very simple example: a binary counter capable of discriminating among the 4 numbers: 00, 01, 10, 11. When the counter says binary “3,” it is not just discriminating 11 from everything else as an undifferentiated bunch; otherwise it would not be a counter, but a 11 detector. To be a counter, the system must be able to tell 11 apart from 00 as well as from 10 as well as from 01 in different, specific ways. It does so, of course, by making choices through its mechanisms, for example: is this the first or the second digit? Is it a 0 or a 1? Each mechanism adds its specific contribution to the discrimination they perform together. Similarly, when we see light, mechanisms in our brain are not just specifying “light” with respect to a bunch of undifferentiated alternatives. Rather, these mechanisms are specifying that light is what it is by virtue of being different, in this and that specific way, from every other alternative. Thus, they specify at once that light is different not only from dark, but also from any color, any shape, any movie frame, any sound or smell, and so on, in every instance in a very specific way. In this way, light acquires its specific *meaning*: light as opposed to dark, not colored as opposed to colored (any color), diffuse as opposed to having a particular shape (any particular one), visual as opposed to auditory or olfactory, sensory as opposed to thought-like, and so on. To us, then, light is much more meaningful precisely because we have mechanisms that can discriminate this particular state of affairs we call “light” against a large number of alternatives.

By contrast, when the photodiode signals light, what does it mean? The photodiode has no mechanism to discriminate colored from achromatic light, even less to tell which particular color the light might be. As a consequence, all light is the same to it, as long as the intensity exceeds a certain threshold. So for the photodiode “light” cannot possibly mean achromatic as opposed to colored, not to mention of which particular color. Also, the photodiode has no mechanism to distinguish between a homogeneous light and a bright shape – any bright shape - on a darker background. So for the photodiode light cannot possibly mean full field as opposed to a shape – any of countless particular shapes. Worse, the photodiode does not even know that it is detecting a visual attribute – the “visualness” of light - as it has no mechanism to tell visual attributes, such as light or dark, from non-visual ones, such as hot and cold, light or heavy, loud or soft, and so on. As far as it knows, the photodiode might just as well be a thermistor – it has no way of knowing whether it is sensing light *vs*. dark or hot *vs*. cold.

In short, generating a large amount of integrated information entails having a highly structured set of mechanisms that allow us to make many nested discriminations (choices) as a single entity. Each of the nested choices is an “*informational relationship*.” According to the IIT, these mechanisms working together generate integrated information by specifying a set of informational relationships that completely and univocally determine the quality of experience.

In the present paper, we set out to characterize mathematically the set of informational relationships generated by a complex of elements. First, we define qualia space (Q) as a space where each point is a probability distribution on the possible states of the system. The informational relationships can then be thought as arrows between points in Q generated by causal mechanisms. We then argue that each experience or *quale* corresponds to a particular set of arrows linking points in Q, that is, an experience is a shape in Q-space. We examine some of the properties of qualia, including entanglement, concepts, and modes. Finally, we show that the language of Q can capture, in principle, some of the basic distinctions that can be made in our own phenomenology, as well as some key neuropsychological observations. Hopefully, this framework can help translate the seemingly ineffable qualitative properties of phenomenology into the language of mathematics.

## Model

In what follows, we consider isolated systems of binary elements, idealizing the silence (0) and firing (1) of neurons. We further assume that elements are memoryless (first order Markov processes) and time passes in discrete instants (e.g. milliseconds). These simplifying assumptions are not essential and will be relaxed in further work. Elements are linked via directed connections and respond to their inputs according to simple Boolean or probabilistic functions, which together constitute the mechanism.

*Notation.* We refer to systems and subsets of systems by capital letters: X, S and so forth. Uppercase letters with subscripts (X_0_, S_0_) denote probability distributions of perturbations that are physically imposed on the outputs of a subset at a given time, e.g. at t = 0. Lowercase letters with subscripts (x_1_, s_1_) denote events: the actual output of the subset in question at a particular time, e.g. at t = 1.

### Integrated information

Before we deal with the quality of consciousness, we must deal with its quantity. According to the IIT, the quantity of consciousness associated with a complex of elements is given by the amount of integrated information it generates [3]. We briefly recall the framework presented in [3], introducing the notions of effective information and integrated information.

#### Information

**Figure 1:**
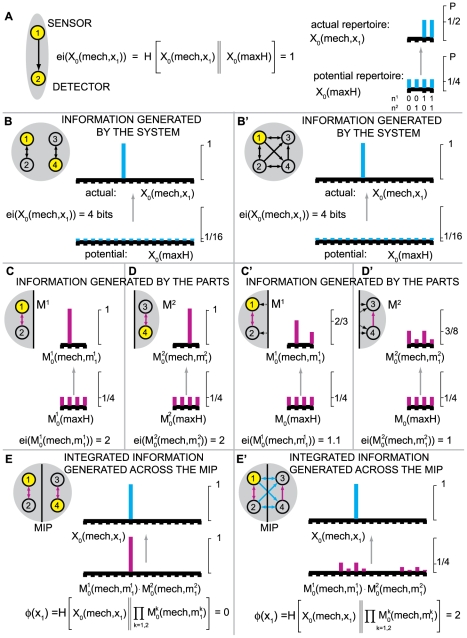
Effective information. (A): A “photodiode” consisting of a sensor and detector unit; the detector unit fires. For the entire system of two units there are four possible states: 00, 10, 01 and 11. The potential repertoire X_0_(maxH) is the maximum entropy distribution on the four states. If the detector fires, its mechanism specifies that the sensor fired at time zero, thus ruling out 2 of the 4 possible states of the system, the actual repertoire is X_0_(mech,x_1_) = (0,0,½,½) on the four states. The prior state of the detector makes no difference to the current state of the system, so the states 01 and 11 are indistinguishable to the mechanism. Relative entropy (also known as Kullback-Leibler divergence) between two probability distributions *p* and *q* is H[*p*∥*q*] = Σ *p*_i_ log_2_(*p*_i_/*q_i_*), so that effective information (entropy of the actual repertoire relative to the potential) is 1 bit. **Integrated information.** *Left-hand side: two double-couples*. (B): the system as a whole generates 4 bits of effective information by specifying that elements n^2^ and n^3^ were on at time t = 0. (CD): The information generated by the system as a whole is completely accounted for by the parts, taken independently. The minimum information partition (MIP) is the decomposition of the system into those (minimal) parts that leave the least information unaccounted for. (E): the actual repertoire of the whole is identical to the combined actual repertoires of the parts (the product of their respective probability distributions), so that relative entropy is zero. The system generates no information above and beyond the parts, so it cannot be considered a single entity. *Right-hand side: an integrated system*. Elements in the system are ON if they receive 2 or more spikes. The system enters state x_1_ = 1000. (B′): the mechanism specifies a unique prior state that causes (leads to) state x_1_, so the system generates 4 bits of effective information. All other perturbations are ruled out since they cause different outputs. (C′D′): effective information generated by the two minimal parts, considered as systems in their own right. External inputs (dotted black arrows) are treated as extrinsic noise. (E′): the information generated by the whole (cyan arrows) over and above the parts (purple arrows). This is computed as the entropy of the actual repertoire of the whole relative to the combined actual repertoires of the parts: Φ(x_1_) = 2 bits. The system generates information above and beyond its parts, so it can be considered a single entity (a complex).

Consider the system in Fig. 1A, meant to represent a binary photodiode. The system has a mechanism such that if the sensor is on (S state = 1), the detector turns on (D state = 1), and is currently in state [11]. How much information is generated by system X, endowed with causal mechanism *mech*, being in the particular state x_1_ = (n^1^_1_n^2^_1_) = [1,1] at time t = 1? Prior to considering its mechanism and current state, the system of two binary elements could have been in any of four possible states ([00],[01],[10],[11]) with equal probability p = ¼. This *potential repertoire* (or “*a priori repertoire*”, [3]) is the maximum entropy (maxent or uniform) distribution, which entails maximum ignorance [4], indicated with X_0_(maxH). The mechanism and current state of the system, however, reduce uncertainty, i.e. generate information, about the previous state of the system (at t = 0). This is because only some previous states (in this case, [10], [11], with equal probability p = ½) could have led to the current system state x_1_ through the mechanism X_0_(mech), while previous states [01],[00] could not (p = 0). In general, mechanism and current state specify an *actual repertoire* (*or* “*a posteriori repertoire*”, [3]) or X_0_(mech,x_1_), the probability distribution expressing how compatible previous system states are with the system's mechanism and current state. The effective information generated by the system is the entropy of the actual repertoire relative to the potential repertoire [3] (also known as Kullback-Leibler divergence [5]):
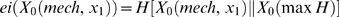



In Fig. 1A, for example, the photodiode generates 1 bit of effective information. Effective information is completely specified the moment the mechanism and the state are specified. In practice, it can be calculated by perturbing the system in all possible ways [6] (all possible input states, corresponding to the maximum entropy distribution or potential repertoire) while keeping track of the resulting actual repertoire using Bayes' rule. Clearly, the amount of effective information generated by the system is high if it has a large potential repertoire and a small actual repertoire. By contrast, effective information is low if the potential repertoire is small (small system) or if the actual repertoire is close to the potential repertoire (for instance, the mechanism is overwhelmed by noise, or many input states lead to the same output states).

#### Integration

Of the information generated, how much is generated by a single entity, as opposed to a collection of independent parts? That is, how much of the information is *integrated information*? Integrated information is measured by comparing the actual repertoire generated by the system as a whole with the combined actual repertoires generated independently by the parts [3]. That is, the actual repertoire for each part is specified by the mechanism internal to each part, considered as a system in its own right, while external inputs are treated as a source of extrinsic noise (Section 1 of Text S1). The comparison is made with the particular decomposition of the system into parts that leaves the least information unaccounted for, called *minimum information partition* (MIP, see [3] for details). Integrated information Φ(x_1_) is then the entropy of the actual repertoire of the system relative to the product of actual repertoires of its minimal parts M^k^ [3].
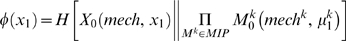



As an example, consider Fig. 1B, representing two of the million photodiodes in a digital camera. By turning on or off depending on its input, each photodiode generates 1 bit of information, just as we saw before. Considered independently, 2 photodiodes generate 2 bits of information, and 1 million photodiodes generate 1 million bits of information. However, as shown in the figure, the product of the actual distributions generated independently by the parts is identical to the actual distribution for the system as a whole. Therefore, the relative entropy between the two distributions is zero: the system generates no integrated information (Φ(x_1_) = 0) above and beyond what is generated by its parts.

Clearly, for integrated information to be high, a system must be connected in such a way that information is generated by causal interactions *among* rather than *within* its parts. Thus, a system can generate integrated information only to the extent that it cannot be decomposed into informationally independent parts. A simple example of such a system is shown in Fig. 1B′. In this case, the interaction between the minimal parts of the system generates information above and beyond what is accounted for by the parts by themselves, and Φ(x_1_) = 2 bits.

In short, integrated information captures the information generated by causal interactions in the whole, over and above the information generated independently by the parts. If a system of elements in state x_1_ generates integrated information Φ>0 and is not contained in some larger set with strictly higher Φ it is called a *complex* (a main complex if its subsets have strictly lower Φ). Indeed, only a complex can be properly considered to form a single entity and thus to generate integrated information.

### Qualia space and qualia

**Figure 2:**
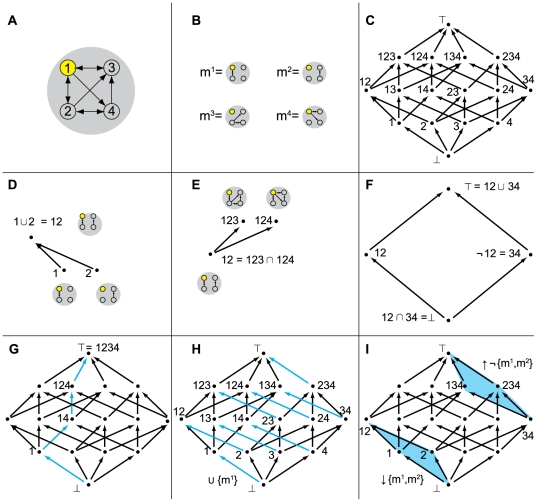
The lattice L of combinations of submechanisms within a system of 4 elements. (A): A system X of 4 elements and 9 connections. (B): Connections in the system are grouped into 4 submechanisms {m^1^, m^2^, m^3^, m^4^} contained within Conn, the set of all connections in X. (C): The lattice of combinations of the 4 submechanisms. (D): The union of submechanisms m^1^ and m^2^ is submechanism m^12^. (E): The intersection of submechanisms m^123^ and m^124^ is submechanism m^12^. (F): The complement of submechanism m^12^ is ¬m^12^ = m^34^. (G): A q-edge is a path from the bottom of the lattice to the top, constructed by engaging each submechanism in sequence. (H): The q-fold generated by all q-arrows of the form r→r∪m^1^, for different contexts r. (I): The down-set ↓{m^1^, m^2^} and the dual up-set ↑¬{m^1^, m^2^}.

To deal with the quality of consciousness, we must consider how the mechanism of a complex specifies an actual repertoire by discriminating a given state (say “light”) not against an undifferentiated bunch of equivalent alternatives, but rather by discriminating that state, in a specific way, against each and every alternative. To do so, we must introduce some tools that serve to characterize the set of informational relationships generated by the mechanism of a complex (Fig. 2).

#### The set of connections Conn

The mechanism of a complex is captured by the set of connections *Conn* among its elements and the rules implemented by the elements. Notation c^ij^ refers to a connection in X from element n^i^ to n^j^. Elements are assumed to implement Boolean or probabilistic functions. A connection between two elements is the minimal meaningful unit of interaction in a system, but connections can mediate interactions among subsets of elements. The system in Fig. 2A (same as in Fig. 1 B′) has 4 elements, and 9 connections among them. A subset m ⊂ Conn is referred to as a *submechanism* of the system, see Section 1 of Text S1. For practical purposes (i.e. to draw the submechanisms on a page), the 9 connections are subdivided into 4 basic submechanisms, Conn = [m^1^,m^2^,m^3^,m^4^], Fig. 2B.

#### The lattice L

The set of all submechanisms of the system, the powerset of Conn, is denoted as the *lattice* L(X), which conveniently captures all possible combinations of causal interactions within X (Fig. 2C). A lattice is a bookkeeping structure that can be used to keep track of inclusion relations, see [7],[8] or Section 2 of Text S1. The *bottom* of the lattice (⊥) is the null set ∅, which contains no connections. The *top* of the lattice (T) contains all connections (hence, T = Conn). Going up the lattice, one encounters all submechanisms: all combinations (subsets) of connections.

The lattice is endowed with three operations: union, intersection and inclusion. Given two members m and r of L, we can form the *union* s = m∪r (the smallest whole containing both, Fig. 2D) and the *intersection* m∩r (the largest part contained in both groups, Fig. 2D). Inclusion of subsets of connections into larger subsets of connections induces a partial ordering on L: if m ⊂ s, then draw an *arrow* m → s. Each subset m of Conn has a unique *complement* ¬m = Conn\m containing all connections not in m (Fig. 2F).

Fig. 2G shows an *edge* in the lattice, which represents a particular path from the bottom to the top. One starts with no connections (bottom), then adds a first submechanism, then two, then three, and finally all of them. Note, this does *not* correspond to travelling through the network over time.

Fig. 2H shows a *fold*. Each cyan arrow is drawn when adding submechanism m^1^ in different *contexts*. For example, the lowest cyan arrow is drawn when considering adding submechanism m^1^ in the *null context*, corresponding to the bottom of the lattice. The next cyan arrow is drawn when considering submechanism m^1^ added in the context of submechanism m^2^. The highest cyan arrow is drawn when considering submechanism m^1^ added in the *full context*, corresponding to all the other connections together ({m^2^,m^3^,m^4^} = ¬{m^1^}), which is where it reaches the top of the lattice. The union of all arrows drawn when adding a particular connection (or submechanism) in all contexts defines the corresponding fold. Thus, all the cyan arrows in Fig. 2H constitute the fold of submechanism m^1^.

Finally, given any subset r of Conn, we can construct two sublattices: the down-set ↓r of all subsets included in r, and the up-set ↑r of all subsets that include r (Fig. 2I).

#### The actual repertoire specified by a submechanism

Each submechanism *m* of *X* discriminates between potential prior states, distinguishing those that cause (lead to) state *x_1_* from those that do not. The discrimination performed by *m* is explicitly described as the *actual repertoire X_0_(m,x_1_)* specified by that submechanism. The actual repertoire is computed by perturbing the system with states in the potential repertoire while using Bayes' rule to keep track of perturbations that cause (lead to) the current state *x_1_*. Connections not in *m* are treated as extrinsic noise and are independently averaged over with the maxent distribution (see Section 1 of Text S1). The empty submechanism ⊥, with all connections disengaged, rules out no alternatives and specifies the potential repertoire X_0_(maxH).

**Figure 3:**
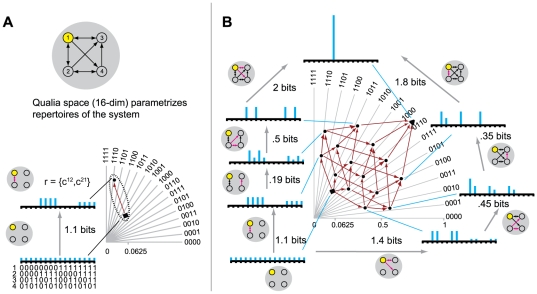
A quale. (A): Qualia space for a system of 4 elements is 16-dimensional (with an axis for each of the 2^4^ possible states of the complex); the axes are flattened onto the page. Upon entering state x_1_ = 1000, the complex generates a quale or shape in Q-space. The quale is generated as follows. The maximum entropy potential repertoire (the “bottom” of the quale) is a point assigning equal probability to all states. Engaging a submechanism (in this case the pair of connections r = {c^12^,c^21^}) “sharpens” the maximum entropy distribution into an actual repertoire, which is another point in Q-space. The q-arrow linking the two distributions (without and with r engaged) geometrically realizes the informational relationship specified by the connections in r. The “length” (divergence) of the q-arrow expresses *how much* the connection sharpens the distribution (the effective information it generates or relative entropy between the two distributions); the direction in Q-space expresses the *particular way* in which the connection sharpens the distribution. (B): Adding additional connections further sharpens the actual repertoire, specifying new points in Q-space and the corresponding q-arrows. The figure shows 16 out of the 399 points in the quale; those generated by combinations of the 4 submechanisms progressively engaged in the insets. The insets around the quale show the repertoires generated along two q-edges (starting at the bottom left, going clockwise and anti-clockwise respectively) formed by q-arrows that engage the 4 sets of connections in two different orders (pink arrows are connections that are engaged; black arrows are connections that have already been engaged in the q-edge). Cyan bars represent probabilities assigned to the 16 possible prior states. Together, the q-edges enclose a shape, the quale, which completely specifies the quality of the experience. Effective information (in bits) of q-arrows in the q-edge is shown alongside.

*Qualia space Q* is a 2^n^ dimensional space (for a system of n binary elements and 2^n^ possible states), having an axis per state and coordinates corresponding to the probability of each state; the space of probability distributions in Q is studied in information geometry [9]. Each submechanism *m* maps to the point in Q given by actual repertoire *X_0_(m,x_1_)*. Fig. 3A shows the mapping of the lattice L into Q for the system shown in Fig. 1B′. Since a 16-dimensional repertoire cannot be drawn, we resort to a 2-dimensional representation of the 16 axes corresponding to the 16 possible states of the system of 4 elements.

*Informational relationships (q-arrows)* represent the “differences that make a difference” [10] to the system, specifically: how discriminations performed by pairs m→m∪r of submechanisms differ (where one submechanism is included in the other). Formally, this intuition is captured as an informational relationship (q-arrow) between two repertoires X_0_(m,x_1_)→X_0_(m∪r,x_1_) in the quale. Informational relationships have counterparts in semantics [11],[12], see Section 5 of Text S1. The “length” (divergence) of the q-arrow expresses the magnitude of the difference between the discriminations performed by the two submechanisms, i.e. the effective information submechanism *r* generates in the context given by submechanism *m*. As before, effective information is the relative entropy between the two repertoires: ei(X_0_(m,x_1_)→X_0_(m∪r,x_1_)) = H[X_0_(m∪r,x_1_)∥X_0_(m,x_1_)]. One can further resolve an informational relationship by considering its internal structure: the shape of sub-lattice ↑m∩↓(m∪r) containing all submechanisms between m and m∪r, see Section 5 of Text S1. In general, the more connections one engages, the more the actual repertoire will differ from the potential repertoire. The entire system (all connections in the complex) specifies the actual repertoire X_0_(T,x_1_), which constitutes the top of the quale. In Fig. 3B this corresponds to a point projecting to p = 1 on one axis and p = 0 on the remaining axes.

*The quale Q(mech,x_1_)* is the mapping of the repertoires generated by all the submechanisms of a complex X into Q; it geometrically unfolds the quality (structure) of the information generated by X. Points of the quale are given by the set of actual repertoires and represent the discriminations made by every submechanism of X. Informational relationships (q-arrows) capture the discrimination performed by a submechanism in the context of other submechanisms (the effective information matrix [1]). The quale can be visualized as a kind of 2^n^-dimensional polytope; its shape completely characterizes *how* the system's mechanism generates information by ruling out alternatives when it enters state x_1_.

*Note*. The quale generated by even a small system is high dimensional, and contains a large number of repertoires and informational relationships. Further, it has a non-metric geometry: effective information (Kullback-Leibler divergence) is not a measure of length. It follows that the quale cannot be *accurately* represented on a flat page. The figures that follow are not “to scale”. Instead, we relate geometric features of interest to important properties of the system and show how they can be quantified.

The quale shows a certain resemblance to graphical models [13]–[15], though there are important differences (see Section 2 of Text S1 for details). A key difference is that in graphical models nodes represent random variables standing for concepts that are taken as given (e.g. RAIN, DANGER) and edges represent conditional dependencies between the given concepts (e.g. p(DANGER|RAIN)). By contrast, in the quale the mechanism and state x_1_ are taken as given. Each point is a perspective provided by a submechanism on the causal interactions that have occurred, and the q-arrows represent how perspectives differ from their subperspectives. A natural question is: How do concepts arise? To answer we must first introduce the notion of entanglement.

### Entanglement

A fundamental property of q-arrows is their entanglement (γ): the extent to which an informational relationship does not reduce to its component relationships (sub-q-arrows). A q-arrow is tangled (γ>0) if its sub-q-qarrows generate information differently taken together than they do taken separately (note the analogy with Φ). As will be described below, entanglement is used to characterize concepts and modes.

**Figure 4:**
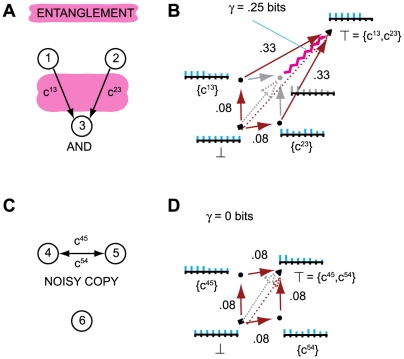
Entanglement. (A): A silent *AND*-gate. (B): The quale generated by the system (maroon arrows). Notice that connections c^13^ and c^23^ generate more information in the full context (.33 bits at the top of the quale) than in the null context (.08 at the bottom). The actual repertoires generated by submechanisms of the system are shown alongside in cyan. Repertoire X_0_({c^13^},x_1_) assigns probability 2/3 to states where n^1^ was silent and 1/3 to states where it was not: the concept “n^1^ probably did not fire”. The actual repertoire of the whole, X_0_({c^13^,c^23^},x_1_), specifies “n^1^ and n^2^ did not *both* fire”, which cannot be recovered from the concepts generated by the two connections taken singly. Entanglement is computed by measuring the entropy of the actual repertoire of the whole relative to the product of the repertoires generated by the two connections singly, shown in gray. (C): A system of three elements, two of which implement the operation *NOISY COPY*: element n^1^ spikes with p = 0 if it receives silent input, and p = ½ if it receives a spike; this is the same operation performed by an *AND*-gate when one of its wires is treated as noise. (D): By construction, the informational relationships generated by connections c^45^ and c^54^ in the null context is the same as connections c^13^ and c^23^ in panel B. However, the qualia generated by the AND-gate and NOISY-COPY system differ because of how the informational relationships tangle at the top of the qualia; an *AND*-gate is not simply the combination of two *NOISY COPY* gates, as can be seen by comparing panels B and D. In the disentangled system, panel D, the actual repertoire of the whole coincides with a product of marginalizations of the actual repertoires of the individual connections.

Fig. 4A shows a tangled informational relationship generated by a silent *AND*-gate. The mechanism of the system, given by T = {c^13^,c^23^}, rules out the prior state [n^1^n^2^] = [11]. As shown in Fig. 4B, the q-arrow X_0_(maxH)→X_0_(T,x_1_) specified by T cannot be reduced to the q-arrows specified by {c^12^} and {c^13^} separately, since the actual repertoire X_0_(T,x_1_) does not reduce to the product of actual repertoires specified by submechanisms {c^13^} and {c^23^} separately. If the sub-q-arrows were not tangled the q-arrow X_0_(maxH)→X_0_(T,x_1_) would reduce to the diagonal of the parallelogram obtained by considering elements n^1^ and n^2^ separately. That is, unentangled q-arrows are orthogonal to each other; while entanglement “warps” the shape of the quale away from a simple parallelogram, see Section 4 of Text S1.

*Entanglement* γ of q-arrow X_0_(m,x_1_)→X_0_(m∪r,x_1_) is the entropy of repertoire X_0_(m∪r,x_1_) relative to the natural decomposition of the q-arrow:
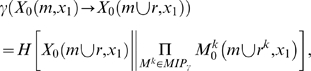
where MIP_γ_ is the minimum information partition for entanglement, by analogy with the formula for Φ. The set r^k^ contains the q-arrows in *r* outgoing from M*^k^*. Thus, entanglement captures how much information a q-arrow generates over and above its natural decomposition into its *minimal q-arrows*. Entanglement of a q-arrow is zero if and only if it decomposes into a collection of independent component q-arrows. The *minimum information partition* for entanglement is found by computing
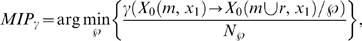
where entanglement for an arbitrary partition is
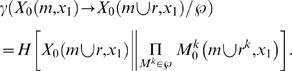



Let R^k^ = src(r^k^) be the source elements for the connections in s^k^. Similar to Φ, the normalization 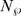 for partition 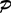 is
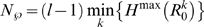
where *l* is the number of parts for which S^k^≠∅. More details on entanglement are provided in Section 4 of Text S1.

*A concept* X_0_(m,x_1_)→X_0_(m∪r,x_1_) is an indivisible informational relationship (γ>0). In other words, a concept is a discrimination performed by some mechanism r in context m that cannot be decomposed into a product of simpler discriminations because the information generated by its constituent sub-q-arrows rely on each other for context. By contrast, a q-arrow with γ = 0 has not internal contextual dependencies, and reduces to its sub-q-arrows without any loss of information. The notion of concept is graded. For a submechanism to generate a concept it must be highly interdependent; for example, a few connections in a system, chosen at random, will (typically) not be tangled.

The simplest concepts are generated by individual connections, which are literally indivisible. The silent AND-gate in Fig. 4A constitutes a simple higher-order concept. Taken separately, the two connection into the silent AND gate generates the elementary concepts {probably not n^1^} and {probably not n^2^}, since in each case maxent noise has been introduced on the other connection. Taken together, however, they tangle and generate the indivisible concept {not both}. The concept {not both} does not reduce to the product of the elementary concepts {probably not n^1^} and {probability not n^2^}, Fig. 4B. Contrast the silent AND gate with the system in Fig. 4C, where elements n^4^ and n^5^ implement the operation *NOISY COPY*, i.e. have the same mechanism as an *AND* gate in which one input connection is given extrinsic noise. The informational relationship generated by c^45^ is the concept {probably not n^4^}, and similarly for c^54^ {probably not n^5^}. In this case, however, the q-arrow generated by both {c^45^, c^54^} is not tangled (γ = 0) as it decomposes into a product of the two smaller, independent q-arrows (Fig. 4D). Thus, the resulting q-arrow means {probably not n^4^ or n^5^}. As such, it does not constitute a single concept, but merely the product of the two independent sub-concepts, and its contribution the quale reduces to that of its components.

*A mode* is a q-arrow that is more densely tangled than its surrounding q-arrows; modes are informational relationships constituting distinct “sub-shapes” in Q. Modes are defined analogously to complexes. Formally, a mode is a maximally dense concept: a mode is an up-set of ¬a, X_0_(¬a,x_1_)→X_0_(mech,x_1_), with γ(X_0_(¬a,x_1_)→X_0_(mech,x_1_))>0 that is not contained in some larger up-set of ¬b, which is (strictly) more densely tangled:
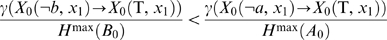
for all b⊃a, where A_0_ contains the source elements in a, and similarly for b. As will be discussed below, modes play an important role in understanding the structure of experience, especially modalities and submodalities. If a mode is contained within a larger mode, we refer to it as a *sub-mode*. By analogy with a main complex, an *elementary mode* is such that its component q-arrows have strictly lower γ.

**Figure 5:**
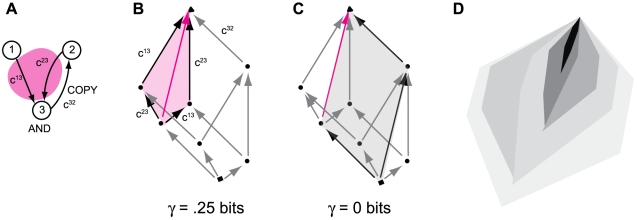
Modes. A mode is a maximally densely tangled q-arrow at the top of the quale. (A): A system containing an *AND* and *COPY* gate. (B): The quale generated by *X*. Connections c^13^ and c^23^ are tangled at the top of the quale with γ = .25 bits. (C): The system as a whole is not tangled: entanglement between connection c^32^ and connections {c^13^,c^23^} is zero. Thus, the up-set ↑¬{c^13^,c^23^} is a mode: it is not contained in a larger up-set with higher γ. (D) Cartoon of a hierarchy of modes in a complex quale.

Fig. 5A shows a system containing an *AND* and *COPY* gate. The *AND*-gate tangles two of the connections in the quale, forming the pink shape in panel B: the concept {not both}. Similarly, the *COPY*-gate generates the concept {not this}. The system as a whole does not generate a single concept, but rather two distinct concepts. This can be seen in panel C where the system as a whole is depicted as a parallelogram, the {not this} and {not both} concepts are orthogonal to one another and do not interact. Since the concept {not both} is not contained in a larger, more densely tangled concept, it forms a mode.

## Results and Discussion

In what follows, we examine some general properties of qualia, and some implications of considering an experience as a shape in qualia space. We also examine how considering basic neurophysiological notions in terms of qualia space affects their interpretation. We then consider some consequences of entanglement and the meaning of concepts, and how learning new concepts affects qualia space. We consider basic examples of how different aspects of phenomenology may be classified as different basic shapes in qualia space, and how, if experiences are shapes in qualia space, they can be compared just as shapes can be. Finally, we consider how a paradigmatic quale, such as “seeing red,” can be thought of in the present framework, with relevant implications for neuropsychology.

For computational reasons, as in [3], we measure integrated information (Φ) and entanglement (γ) by considering all bipartitions and the total partition, instead of all partitions. Further, when measuring entanglement we restrict attention to submechanisms given by connections sourcing from particular elements (rather than arbitrary groups of connections) and measure the entanglement of the bits in those source elements.

### Some general properties of qualia

We first consider some basic results that can be obtained by treating qualia as shapes specified by sets of informational relationships. We show that the amount of integrated information generated by a complex can be interpreted as the “height” of the quale. It also follows that only informational relationships generated within a complex contribute to the shape of the corresponding quale. Another consequence is that the state of a complex is meaningless without considering its mechanism. An intriguing corollary is that different systems in different states may generate exactly the same quale.

#### The quantity of consciousness (Φ) is the “height” of the quale

How does the shape of the quale *Q(mech,x_1_)* reflect the integrated information Φ(x_1_) generated by a system? To address this question, one must consider how partitions, including the minimum information partition (MIP), can be represented in qualia space. Recall that each submechanism specifies an actual repertoire by discriminating between potential prior states. Actual repertoire X_0_(m,x_1_) can be interpreted as that submechanism's perspective on the discriminations performed by the entire system. Each partition is just a point in Q; for example, suppose we have a partition P. For each part M^k^ in P, let the intra-set of M^k^ be all connections for which the source and the target are elements in M^k^. Let P ⊂ Conn (we reuse the symbol) be the union of the intra-sets across the parts in P (the partition): it contains all connections within each part, and no connections between parts. In this way partitions are mapped into L(X):
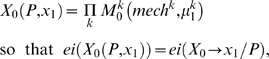
where the right-hand side of each equation uses the notation of [3]. Given partition P, define the extra-set of P to be connections between parts, thus the extra-set of P is the complement ¬P. Effective information ei(X_0_(P,x_1_)) is then the information generated by the extra-set of P (since mech = T = P∪¬P), over and above the intra-set of P. The relations between partition-repertoires inside Q geometrically realize the interactions amongst parts that are performed by the various extra-sets of connections in X. The additional points in Q given by other submechanisms reveal the finer structure of the discriminations performed by the system.

**Figure 6:**
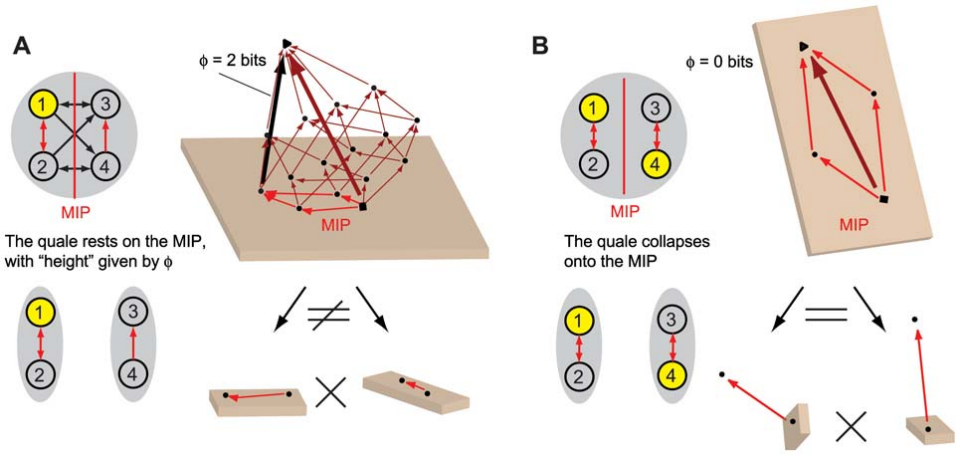
The relationship between qualia and Φ. (A): The quale generated by the system in Fig. 3. The down-set ↓MIP of the minimum information partition forms a natural “base” for the complex. The informational relationships *among* the parts are built on top of the informational relationships generated *within* the minimal parts. From this perspective the Φ q-arrow (in black) represents the “height” of the quale above its base; the “length” (divergence) of the Φ-q-arrow expresses the breathing room in the system. (B): The quale generated by the pair of couples in Fig. 1B. Although the system generates the same amount of effective information and the same actual repertoire (as a whole) as the system in panel A, it does not do so as a single entity. The system breaks into two independent components (the down-set ↓MIP contains the entire quale). The system reduces to its MIP (base); integrated information Φ = 0 so there is no breathing room and no experience is generated. The system breaks into two disjoint components, each of which forms a complex with Φ>0.

The minimum information partition (MIP) is thus just another point in Q, the one specified by the connections within the minimal parts only. The q-arrow X_0_(P^MIP^,x_1_)→X_0_(T,x_1_) has divergence:
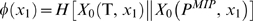



Therefore, Φ quantifies the difference between the perspective provided by the entire system and that provided by the MIP, the partition that *most closely* accounts for the perspective of the whole. For this reason the down-set ↓P^MIP^, which unfolds the structure (or quality) of the perspective provided by the MIP, can be thought of the system's natural information-theoretic “base”. The Φ-q-arrow can then be thought of as the height of the solid, quantifying how much the complex rises above a collection of independent parts. Fig. 6A shows the quale generated by the integrated system of Fig. 2B and 3B (in state 1000). In the figure, the quale has been rotated to rest on this base. In general, the higher the Φ value of a complex, the more “breathing room” there is for the various informational relationships within the complex (the edges of the solid) to express themselves. Alternative geometric methods for decomposing a probability distribution into orthogonal components are developed in [16]–[18]; see Section 3 of Text S1 for a comparison of the approaches and their motivations.

Consider now Fig. 6B. For the double couple of Fig. 2A, which is clearly made up of two disjoint complexes, the entire quale collapses onto sublattice ↓P^MIP^: the actual repertoire of the whole collapses onto its base (MIP), and Φ is zero. The solid is flat: looking at the quale from the natural perspective, level with the ground, nothing is visible. The perspective provided by the MIP completely accounts for the discriminations performed by the system. Indeed, there exists no complex corresponding to the double couple – as a whole, such a system does not generate any quantity of consciousness (measured by integrated information, Φ), nor any quality (measured by the shape of the quale Q). Informationally, and phenomenologically, it does not exist. What exists, instead, are two smaller complexes, each corresponding to a couple of elements joined by a mechanism - for example, two separate photodiodes. Each of them generates a small quale, corresponding to a single q-arrow with no further structure.

The systems shown in Fig. 6AB are idealized examples of an integrated and a strongly modular system respectively. Prior work [3] has shown that modular systems – such as the cerebellum – typically generate low Φ. Although we cannot draw the qualia of large modular systems, the figure shows how the quale of a system with low Φ lies low on its base, which is given by the partition into near-independent modules. As a system becomes more functionally integrated, whilst remaining functionally specialized, Φ increases as the system becomes less a collection of independent parts, and more a single entity.

#### Only informational relationships within a complex are part of the same quale

If experience is integrated information within a complex, it follows that only the informational relationships within a complex contribute to experience [1]. Conversely, the informational relationships that exist outside a main complex – for example those generated in a separate complex, or those involving sensory afferents or cortico-subcortical loops implementing informationally insulated subroutines [1] – cannot contribute either to the quantity or to the quality of consciousness generated by the main complex. As illustrated in Fig. 6B, though one may attempt to draw the quale generated by a collection of n = 4 elements forming two separate complexes in the full 2^n^ = 16 dimensional qualia space, it turns out, upon closer inspection, that its shape does not exist in the full-dimensional space (the solid is flat). Rather, the shape collapses into two simpler qualia living in lower-dimensional qualia spaces (2^2^ = 4-dimensional), one per complex. In the case of overlapping complexes, informational relationships specified by the same mechanism may live in different qualia, a higher–dimensional one corresponding to the main complex, and a lower–dimensional one corresponding to a larger complex of lower Φ. In summary, only the informational relationships within a complex contribute to giving the quale its shape.

#### The same system in different states may generate different qualia

**Figure 7:**
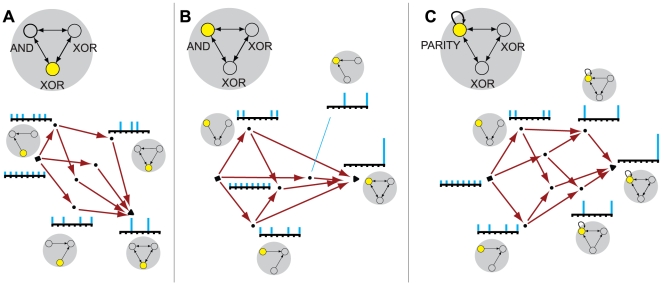
The quale depends on the mechanism and the state. (AB): The same system (an *AND*-gate and two *XOR*-gates) in two different states generates two different qualia, two different experiences. (BC): Two systems in the same state, but with different mechanisms, generate different qualia.

When the same system (mechanism) is in a different state (firing pattern), it will typically generate a different quale or shape, even for the same value of Φ. Figure 7AB show the same system (a simple AND/XOR system) in two different states (x_1_ = 001,100). Since the connections are engaged in different ways when the system is in two different states, the interactions within the systems are qualitatively different. As shown in Fig. 6, systems sharing the same actual repertoire as a whole may also generate different qualia since their submechanisms generate information differently.

#### Different systems in the same state may generate different qualia

A quale is specified by a mechanism and a particular state - it does not make sense to ask about the quale generated by a mechanism in isolation, or by a state (firing pattern) in isolation. A consequence is that different systems in the same state can generate different qualia. Fig. 7BC shows two systems, the AND/XOR system and the PARITY/XOR system. The two systems are in the same state (x_1_ = 100); and both generate Φ = 3 bits; in both cases the minimum information partition is the total partition (MIP = 123|∅). However, the two systems differ both in their connectivity and in the rules that the elements implement, so the quale generated by the AND/XOR-triple is structured differently from the PARITY/XOR-system. As an extreme example, a system that were to copy one by one the state of the neurons in a human brain, but had no internal connections of its own, would generate no consciousness and no quale [1],[3]. Thus, the notion of state is meaningless without taking into account the mechanism that produces the state.

#### Different systems in different states may generate the same quale

**Figure 8:**
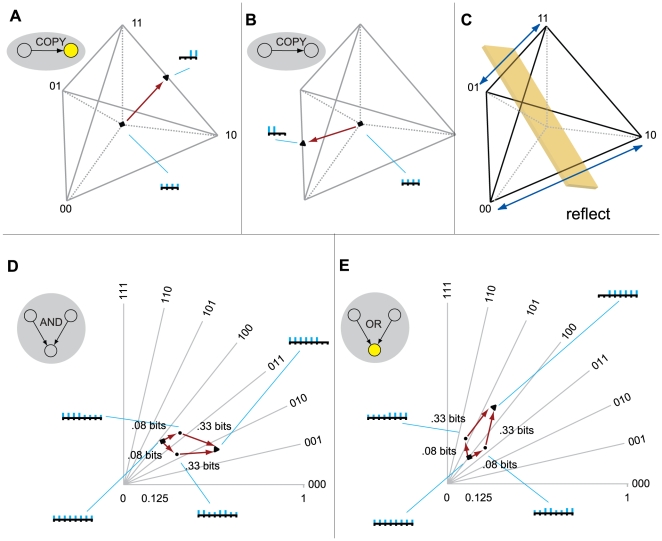
Isomorphisms between qualia. (A): The simplest possible system: a sensor and a detector, where the detector copies the prior state of the sensor. The quale generated by the system when the detector is ON is a single q-arrow with effective information of 1 bit. The q-arrow specifies the sensor was ON in the previous time step. (B) When the detector is OFF, the system generates a different quale, where the q-arrow points in a different direction – towards a different actual repertoire – specifying that the detector was OFF. Effective information is again 1 bit. (C): A reflection of Q-space generated by relabeling the outputs of n^1^ (flipping 0 and 1) induces an isomorphism between the two qualia. (DE): The qualia generated by a silent *AND*-gate and a firing *OR*-gate respectively. The two qualia are isomorphic, which can be seen by flipping the roles of 0 and 1.

By the same token, it is possible that two different systems generate the same quale. As an example, consider again the photodiode, whose mechanism determines that if the current in the sensor exceed a threshold, the detector turns on. Informationally, the photodiode implements a COPY system, where the detector copies the state of the sensor. This simple causal interaction is all there is, and when the photodiode turns on it merely specifies an actual repertoire where states (x_1_ = 00,01,10,11) have, respectively, probability (0,0,½,½) (Fig. 8A). This corresponds in Q to a single q-arrow, one bit long, going from the potential, maximum entropy repertoire (¼,¼,¼,¼) to (0,0,½,½). Now imagine the light sensor is substituted by a temperature sensor with the same threshold and dynamic range - we have a thermistor rather than a photodiode, and assume that the detector is off (low temperature, Fig. 8B). While the physical device has changed, and its state is different, according to the IIT the experience, minimal as it is, has to be the same, since the informational relationship that is generated by the two devices is identical.

#### Qualia isomorphism

The symbols 0 (off) and 1 (on) are arbitrary labels given to interchangeable outputs. In fact, there is an *isomorphism* between the two qualia: the reflection in Fig. 8C relabels the outputs of n^1^, flipping 1 and 0. Thus, a binary device like a photodiode or a thermistor generates the same qualia regardless of the state it is in; the two qualia are equivalent. The system is memoryless, so every input is a surprise (even if they are all the same); to be a binary photodiode or thermistor is to rule two out of four potential states at each instant. As can be seen from the quale, there is no additional structure to the system. An isomorphism between two qualia is an identification of the qualia spaces, Q(X) and Q(Y), by relabeling elements and outputs, that induces a lattice isomorphism from Q(x_1_) onto Q(y_1_). A lattice isomorphism is a bijection preserving the lattice structure, see Section 2 of Text S1.

As another example, consider the qualia generated by a silent AND-gate and by a firing OR-gate (Fig. 8DE). Comparing panel D with panel E, it is apparent that relabeling the outputs of the top two elements produces an isomorphism between the qualia generated by a silent AND-gate and a firing OR-gate (it is easy to show that the converse is also true: the qualia generated by a firing AND-gate and a silent OR-gate are isomorphic). Thus, in simple systems it is possible that symmetries and isomorphisms may lead to different physical systems generating the same quale [19]. As a consequence, to be a silent AND-gate is indistinguishable from being a silent OR-gate; similarly, to be a COPY-system, in any state, is indistinguishable from being a NOT-system, in any state (symmetries in a more interesting example, the AND-triple, and in a parity system, are analyzed in Section 7 of Text S1). It should be kept in mind, however, that in more complicated systems symmetries are likely to break. Thus, it is extremely unlikely that two different biological systems would generate identical experiences.

### Some implications for neurophysiology

Considering the information generated by a complex of elements in terms of the shape they specify in Q has some implications for the way we interpret neurophysiologic data. Rather than trying to understand the meaning of the activity of some elements (neurons) in isolation, or even of distributed patterns of activity or of correlations, the IIT claims that meaning is only generated in terms of shapes in Q, that is, in terms of the set of informational relationships generated by a complex. Below we examine a few representative examples that clarify the perspective provided by the IIT. For instance, we show that, in Q, the same connections can specify different informational relationships in different contexts. Next, we show that removing a set of connections (mimicking a lesion) simplifies the shape of the quale by collapsing it along a q-fold. We also illustrate how, when an element (a neuron) turns on, it generates information by changing the shape of the quale. Moreover, informational relationships, and thus the shape of the quale, are specified both by the elements that are firing and by those that are not. Finally, “inactivating” elements that are already inactive has major consequences on the shape of the quale, though the firing pattern remains the same.

#### The same mechanism can generate different informational relationships in different contexts

**Figure 9:**
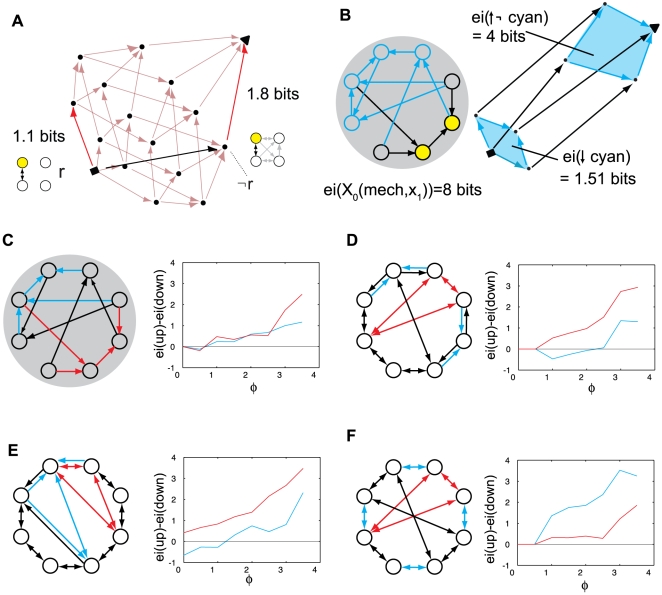
Context-dependency of informational relationships. (A): The same set of connections engaged in two different contexts (red arrows) for the system in Fig. 3. At the bottom of the quale (in the null context) the connections generate 1.1 bits of information, whereas the up-set of the connections, in the full context, generates 1.8 bits of information. (B): A system of *AND*-gates. The four cyan elements generate 1.5 bits of information in the null context and 4 bits of information in the full context. (CDEF): The relationship between Φ and context-dependence. Each panel shows a system of 8 *AND*-gates with two sets of connections chosen, shown in red and cyan (in panel E a connection is chosen twice). Each point in the graphs shows the average value of the difference: “r in full context – r in null context” = ei(X_0_(¬r,x_1_)→X_0_(T,x_1_))−ei(X_0_(maxH)→X_0_(r,x_1_)), averaged across network states where Φ is in the range [k,k+0.5), as k varies from 0 to 3.5 bits. The graphs show that, context as Φ increases, the information generated by a set of connections in the full context increases relative to the same connections in the null.

Informational relationships are context-dependent, in the following sense. Recall from the Model section that a *context* is a point in the lattice L corresponding to a particular submechanism m. In Q, this point corresponds to the actual repertoire generated by that submechanism. As shown in Fig. 9A, the q-arrow generated by a connection (how it further sharpens the actual repertoire) can change in both magnitude and direction depending on the context. In Fig. 9A, when considered in isolation (null context), the connection *r* between elements 1 and 2 generates a q-arrow of 1.1 bits pointing in a certain direction. When considered in the full context provided by all other connections (¬r), the same connection *r* generates a longer q-arrow (1.8 bits) pointing in a different direction. Another example is shown in Fig. 9B, a system of 8 *AND*-gates. The four cyan elements generate 1.5 bits of information in the null context and 4 bits of information in the full context, and the informational relationships point in different directions. Panels CDEF show results averaged across many different states of the same system, for different submechanisms. The results show that the information generated by a set of connections is higher in the full context than in the null context when a system generates high Φ. Thus, within an integrated system, a submechanism produces different informational effects in different contexts, and usually it produces larger effects the richer the context. Note that this result is fully compatible with empirical work on functional connectivity [20] and related theoretical considerations [21],[22] on the role of neural context in cognition.

#### Lesioning a mechanism collapses a quale along a q-fold

**Figure 10:**
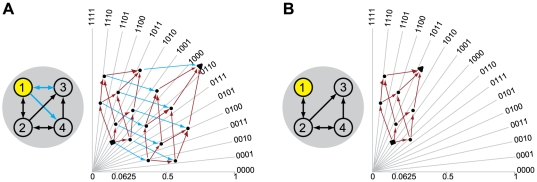
Collapse of a q-fold. (A): The quale generated by the system in Fig 3. (B): The connections in cyan are removed and replaced with noise. The quale collapses onto a subquale.

Removing a set of connections from every possible context *folds* a quale. As shown in Fig. 10, if we remove the submechanism r from the system (same as in Fig. 3), all the q-arrows generated by that connection, in all possible contexts, vanish, so the shape of the quale “folds” (collapses) along the dimensions specified by that connection. Conversely, when the connection is added to a system, the shape of the quale *unfolds*. Thus, within an integrated system, a connection produces informational effects in many different contexts, and these effects can be captured precisely by changes in the shape of the quale along a fold.

#### When an element within a complex becomes active, it changes the shape of the quale

**Figure 11:**
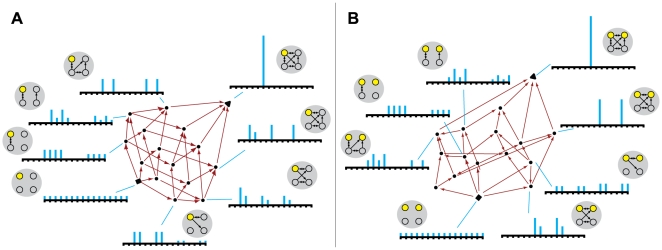
When an element becomes active, it changes the shape of the quale. (A): the quale generated by the system in Fig. 3, when x_1_ = 1000. (B): If element n^3^ becomes active, changing the firing pattern to x_1_ = 1010, the quale changes shape. The firing of an additional element changes almost all of the actual repertoires (see insets).

In neurophysiology, one often searches for neurons that fire for particular inputs. It is often assumed that, when such neurons fire, they “broadcast” the relevant information to a large public of other neurons [23]. However, it is hard to see how the firing of a neuron may convey the meaning of those inputs, when all it can do is fire or not. A similar problem obtains for the neuron receiving its output. Each of them may receive up to 10,000 input lines, some firing, some not. How is a target neuron going to know that one of its input spikes means “red” or a particular shape? According to the IIT, what matters is that, within a complex, the firing of a neuron that was previously off changes the shape of the entire quale, which is what carries the meaning. As a simple example, consider the complex in Fig. 11 (same as in Fig. 3). Assume, for instance, that element n^1^ stands for a neuron selective for a “square” shape, which is currently firing due to the presence of a gray square in the visual field (Fig. 11A). Now assume that the square turns red and another neuron (n^3^), which was silent, becomes active (Fig. 11B); integrated information is 2 bits for both activity patterns. Clearly, the activation of element n^3^ changes the shape of the quale, since it modifies almost all of the actual repertoires (insets). From the extrinsic perspective of a neurophysiologist, if the n^3^ neuron became active every time a subject reports seeing red, it is natural to label the activation of the “red” neuron n^3^ as the neural correlate of consciousness for red [24]. From the intrinsic perspective of the complex, however, the meaning of “red” can only be realized by a change in the shape of the quale triggered by the firing of the red neuron. As shall be further discussed below, the NCC for red cannot be captured by the firing of a particular set of neurons, or even of larger circuits, but only by a particular shape in Q.

#### Inactive elements specify the shape of a quale

The assumption that neural elements that are active are broadcasting information often goes hand in hand with the corollary that inactive elements are essentially doing nothing, since they are not broadcasting anything. According to the IIT, this is not correct. In the general case, being “off” is just as informative as being “on.” An element that fires specifies previous states that would have made it fire and rules out other states. Similarly, an element that does not fire rules out previous states of affairs that would have made it fire and thereby contributes to specifying the actual repertoire. For example, in Fig. 9B silent elements generate 4 bits information.

**Figure 12:**
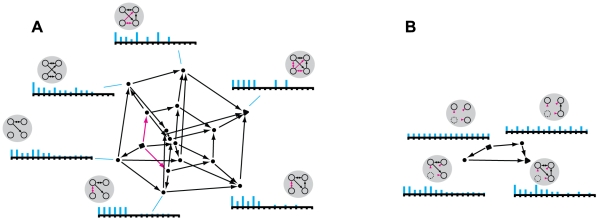
Inactive versus inactivated elements. (A): The quale generated by the system in Fig. 3 when no elements are firing. The shape is not drawn to scale, and is considerably smaller than that generated for x_1_ = 1000 or 1010: effective information of the whole ei(X_0_(maxH)→X_0_(T,x_1_)) = 1.2 bits, as opposed to 4 bits when element n^1^ is spiking. The actual repertoire of the whole is not specified precisely. (B): By contrast, if element n^2^ is inactivated – rather than merely inactive – and connections with source n^2^ are replaced with noise, the quale collapses.

In a neurophysiologic context, constraints such as energy costs may dictate that being “on” should be used more sparingly than being “off.” In that case, a system should reserve firing for states of affairs that are less frequent and therefore more informative (Balduzzi and Tononi, in preparation), so values of Φ may not be as high when all elements are silent compared to when an adequate fraction are active [3]. Even so, inactive elements remain informative, and jointly they can rule out a vast number of previous states. Indeed, a complex with no elements firing can generate a quale with a non-trivial shape. Fig. 12A show such a system, the same as in Fig. 3 and 11, but with all elements inactive. The quale generated by a complex with all elements inactive may be considered as the “*default*” quale. A default quale has the prerogative of expressing geometrically all relationships implemented by the system's mechanisms, without weighing any mechanism more than any other. Whether the brain can sustain for sufficient periods of time a state in which no neurons are firing (or they are all firing at a baseline rate expressing readiness but not true activation), remains to be determined. Possibly such a state may be reached in certain meditation practices, and may correspond to a state of full consciousness with no particular content [25].

#### There is a difference between inactive vs. inactivated elements

As shown in the previous examples, inactive (i.e. silent) elements generate information and thus contribute to specifying the shape of the quale. Along the same lines, a somewhat counterintuitive prediction stemming from the IIT is that if elements within a complex are inactivated, rather than merely being inactive, experience should change, although the firing pattern is the same. Consider again Fig. 12A, where 4 inactive elements generate a default quale. In Fig. 12B, element n^2^ is not merely inactive, but it has been inactivated, meaning that its mechanism has been disabled and the connections with source n^2^ have been replaced with noise. It is evident that, despite the identical “firing pattern,” the quale in Fig. 12B collapsed, shrinking dramatically in both quantity and quality. Once again, what matters is the set of informational relationships (the shape of the quale) generated by a given mechanism and firing pattern together [1],[3].

### Concepts and learning

An informational relationships is tangled if it does not reduce to its component relationships, see above. As introduced in the Model section, connections considered together can generate information above and beyond the information they generate separately. Entanglement, which is used to define concepts and modes, characterizes informational relationships (q-arrows) that are more than the sum of their component relationships (Fig. 4). Below we consider the informational advantages of entanglement. We will also consider how learning can generate new concepts, leading to more differentiated qualia. The next section will consider modes.

#### Concepts

**Figure 13:**
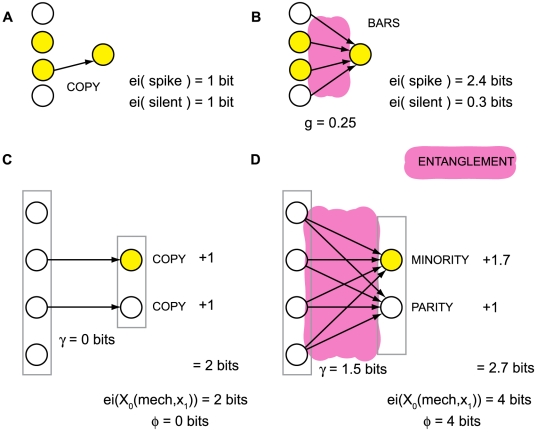
Tangled concepts can generate more information about their inputs than their atomic subconcepts. (A): An element extracts information from a set of four sensors. If the input received by the sensor layer is pure noise (the maximum entropy distribution on 2^4^ = 16 possible firing patterns) then the best a single element can do, on average, is to extract 1 bit of information. An efficient strategy is to *COPY* the output of one of the sensors, so that the element generates a concept of the form *ON/OFF*. (B): An element that spikes if it receives a *BAR*: 1100, 0110 or 0011. If a bar is presented, the 4 connections together generate 2.4 bits of information, whereas the individual connections generate 0.08 bits independently. For the 4 connections to generate more information as a whole than separately they must be tangled: γ = 0.25 bits. If the input pattern is not a bar, the element generates 0.3 bits, so that it performs worse than the *COPY*, on average, on maxent noise. However, if bars are sufficiently common in the input, then the element generates more information than a *COPY* element. (C): Two elements *COPY* their inputs. This produces the maximum possible average effective information (2 bits for 2 binary elements) assuming the inputs are maxent distributed. The elements are not tangled, γ = 0, and so the whole generates information equal to the sum of the parts. (D): A cartoon cortical area: a subsystem that receives more inputs than there are elements. If there is some statistical structure to the inputs (certain patterns are more common than others), the system can form concepts specific to the input structure. The 2 binary elements shown generate 4 bits of information about the input pattern, more than the elements taken individually (2.7 bits). On average, using maxent, the 2 elements generate 1.8 bits, less than the *COPY* elements. However, if the inputs are structured and so not maxent, the elements can generate more information about other cortical areas than they should “by right” by tangling informational relationships into concepts and modes.

Figure 13A shows a system comprising 4 input elements (sensors) and 1 output element (detector), which implements a *COPY* of one input element. In doing so, the COPY element generates 1 bit of information, whether it fires or not, and specifies a single informational relationship (q-arrow), corresponding to the simplest possible concept: that things are one way rather than another way, just like the photodiode in Fig. 1. If the input is pure noise (the maximum entropy distribution on 2^4^ = 16 possible input patterns), then extracting 1 bit of information is indeed the best a single element can do. By contrast, the “*BAR*” element in Fig. 13B “integrates” information from 4 sensors. If the input is 1100, 0110, or 0011, the BAR element fires, and generates 2.4 bits of information, more than the COPY element. It can do so because the connections it receives from the 4 sensors are tangled, meaning that jointly they generate more information than the sum of the information generated by each connection independently (0.08 bits each). The corresponding tangled informational relationship (γ = 0.25 bits) corresponds to the concept BAR. By contrast, when the input pattern is not a bar (13 patterns out of 16), the element generates 0.3 bits. On average, then, the BAR element performs worse than the COPY element on pure noise, but can do better, thanks to entanglement, if bars are a common statistical feature of the input, i.e. more common than other patterns. In general, “integrating” information through entanglement and the formation of concepts is an effective strategy to extract more information from an input under the constraint of dimensionality reduction (here from 4 inputs to 1 output), as long as the input has some statistical structure. Neurons are certainly well-suited to extracting information from their input [26], and they must perform extreme dimensionality reduction, as they receive thousands of inputs but emit a single output. Indeed, it is frequently stated that neurons are wired to “integrate information.” The notion of entanglement provides a precise formulation of this function.

Consider now multiple detector elements. In Fig. 13C, 2 elements copy their respective input. Again, if the inputs are distributed with maximum entropy, 2 independent COPY systems generate the maximum possible average effective information, whether they fire or not (2 bits for 2 binary elements). The 2 connections are not tangled (γ = 0), as the information they generate jointly is equal to the sum of the information they generate independently. In the *CONCEPTUAL* system (Fig. 13D), each of 2 detector elements integrates information from 4 sensors, just as in Fig. 13B. Again, each CONCEPTUAL element can do better than a COPY element if there is statistical structure to the inputs. For example, the MINORITY element generates 1.7 bits. Note also that, since the 2 detector elements in Fig. 13D specify different concepts (MINORITY and PARITY), they generate information about different aspects of the input string. Indeed, jointly the 2 CONCEPTUAL elements generate more information (4 bits) than independently (1.7+1 = 2.7 bits), so their afferent connections must be tangled (γ = 1.5 bits), see Section 4 of Text S1. Since it is tangled, the CONCEPTUAL system as a whole can generate more information than a COPY system under the constraint of dimensionality reduction, as long as there is matching statistical structure in the inputs. In future work, we will relate the concepts generated by a system to Bayesian inference [27]–[29].

This simple example also illustrates the importance of considering integrated information as opposed to just (effective) information. As previously shown (Fig. 1B and 6B), the COPY system is a collection of 2 independent parts, each generating 1 bit of integrated information. From an extrinsic perspective, the COPY system transmits information effectively, in this case the 2 bits corresponding to the 2 sensors. However, from an intrinsic perspective, there is no single entity that “knows” the state of both sensors Φ = 0 bits; there are instead 2 independent systems, each of which “knows” 1 bit about its respective sensor (see Text S1, Section 1). By contrast, the CONCEPTUAL system *integrates* information (Φ = 4 bits): it constitutes a complex that knows both inputs as a single entity, above and beyond what its parts know. Specifically, in this case the complex knows that the entire sensor layer is silent, since it knows at once that the input is <2 and even. Integrating information would seem to be an advantage for organisms that need to make unified decisions that are sensitive to context. As shown in Section 4 of Text S1, entanglement ensures that elements are part of a complex, and thus that Φ>0 bits.

#### Qualia can become more complex by learning new concepts

Experiences can be refined through learning and changes in connectivity [30]–[33]. Say one learns to distinguish wine from water, then red from white and rosé wines, then different varietals. Presumably, underlying this phenomenological refinement is a neurobiological refinement: neurons that initially were connected indiscriminately to the same afferents, become more specialized and split into sub-groups with partially segregated afferents. This process has a straightforward equivalent in Q: the single q-arrow generated initially by those afferents splits into two or more q-arrows pointing in different directions, and the overall sub-shape of the quale becomes increasingly complex.

**Figure 14:**
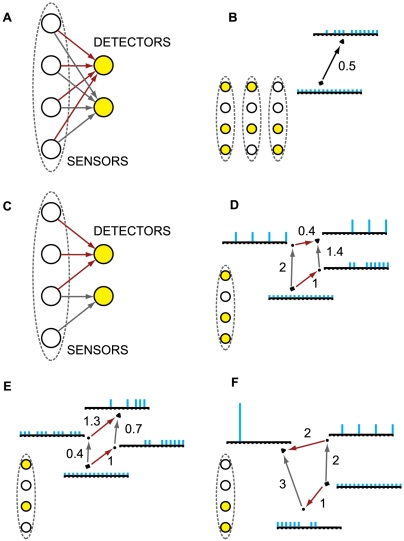
Learning to distinguish new experiences enriches the shape of the quale generated by a system. (A): A system of elements, containing two detectors (*AND*-gates that respond to >1 spike) and four sensors, on which we focus attention. The sensors have all-to-all connections with the detectors. Both detectors are firing, which occurs for any of the sensor patterns 1011, 1010 and 0011 (amongst others): “wine”. (B): The quale generated by the system. The maroon and gray submechanisms (containing 4 connections targeting each detector) generate a *single* q-arrow due to the redundancy of the all-to-all connectivity. The system generates the same quale in response to three different sensor patterns: “rosé wine” (1011), “red wine” (1010) and “white wine” (0011). (C): The system learns to distinguish between types of wine by pruning three connections; as before detectors are *AND*-gates, however, since their inputs differ they are no longer redundant. (DEF): The three sensor patterns generate three different qualia. Moreover, each quale is richer than in panel B: the single q-arrow has split into 4 q-arrows, reflecting the increased richness in *how* the taste of different wines is specified.

Fig. 14A shows the quale generated by a system where 2 detector elements receive identical connections from all 4 sensors. For 3 different input patterns (say rosé, red, and white wines) the responses of the detectors is the same: both elements are firing, indicating the detection of wine as opposed to water (in which case they would be silent). The quale reflects the redundancy of the concepts generated by the elements: the 2 submechanisms consisting of connections targeting the two detectors are redundant and generate a single q-arrow in the quale onto which all 3 wine patterns collapse: the experience is an undifferentiated one of wine (as opposed to water; we are assuming here that the quale is much larger than what is actually drawn, including all the context necessary to specify that these are gustatory experiences having to do with liquids).

Suppose that learning the difference between red and white wine causes the detectors to become specialized by pruning some connections (Fig. 14C). Since the 2 elements have different mechanisms (in this case, they receive from different subsets of sensors, and thus specify 2 different concepts), the information they generate is no longer redundant. As a consequence, the shape of the quale becomes more complex, even for exactly the same firing pattern. Indeed, when both detectors are firing, the shape encodes “rosé” as opposed to red or white, each of which would give rise to a different shape. Thus, with learning experience becomes more differentiated, and this differentiation is reflected in an increased complexity of the shape of the underlying qualia.

### Modes

In the Model section, *modes* were defined, by analogy with complexes, as q-arrows that are more densely tangled than surrounding q-arrows. Whether a complex consists of a single mode or of multiple modes and submodes depends as usual on both its connectivity (mechanism) and activity pattern. In what follows we argue that the subdivision of experience into modalities and submodalities corresponds to sub-shapes (modes) in Q. Moreover, we argue that qualia in the narrow sense are elementary modes (not further decomposable); and that homogeneous/composite experiences are homogeneous/composite shapes.

#### Modes and submodes are a function of both connectivity and activity patterns

**Figure 15:**
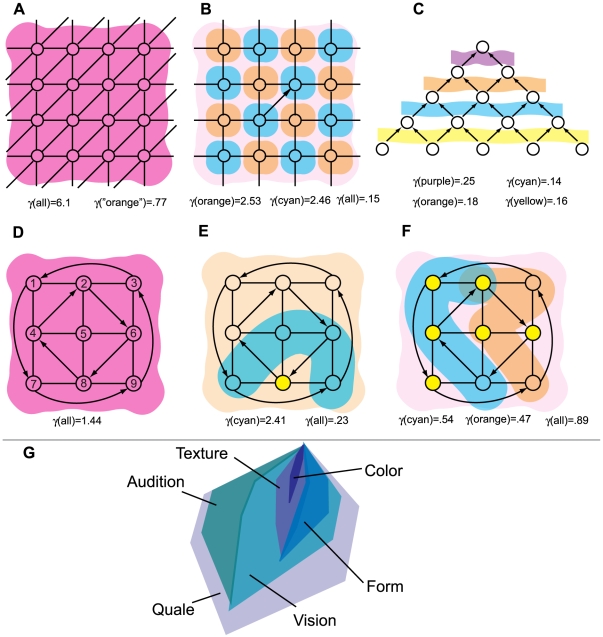
Modes depend on network structure and network activity. Elements in all panels are AND-gates firing if they receive 2 or more spikes. Lines represent bidirectional arrows. (ABC): Modes and network structure. (A): A honeycomb grid (with bidirectional connections and torus edges) generates a single mode. γ(“orange”) computes entanglement for the elements colored orange in panel B. (B): Removing most of the diagonal connections, results in a system containing two weakly tangled modes, shown in cyan and orange, arranged in a chessboard-pattern. The single diagonal connection loosely tangles the two modes. (C): A diagonal slice of a feedforward grid. Each layer of the grid is a separate mode, disentangled from the others. (DEF): Modes and network activity. (D): “Nothingness”. A silent system forms a single, homogeneous mode. (E): “Pure red”. The system as a whole forms a weak mode (orange). The strongest mode (cyan) is created by the firing of a single element. (F): “A composite experience”. A more complex firing pattern results in multiple overlapping modes, two of which are shown. (G): A 2D cartoon of modes in a quale. At the top is the color mode. Currently, the system is exposed to a red stimulus, so the informational relationships within the mode specify the redness of red: the direction of the q-arrows within the mode – and how they are tangled – is what makes red different from green or blue. However, the context afforded to red – the fact that it is a visual rather than auditory experience – is not a property of the color mode. The color mode is contained in a series of larger modes: form, vision, perception, which fill in the context in which the redness of red is specified. The vision mode as a whole is a tangled concept, which cannot be decomposed into independent subconcepts, even though the submodes, such as color and motion, have a certain amount of independence. Color is always associated with a shape of some kind (a totally red visual field is a particular shape), and also motion (awareness of lack of motion is awareness of a kind of motion), and so forth. The quale of the entire system itself forms a mode since γ>0.

Figure 15A shows a complex made up of AND-gates (here each AND has six afferents) that constitutes a single mode, indicated as a single pink blob having γ = 6.1. Eliminating certain connections gives rise to two separate modes, indicated as neighboring cyan (γ = 2.46) and orange blobs (γ = 2.53). However, the system still forms a single complex, and indeed there is a larger, albeit weaker, mode encompassing all connections with γ = 0.15. In Fig. 15C, eliminating other connections gives rise to 4 separate modes. In this case, the complex does not form a single mode (γ = 0). Thus, the quale or shape generated by a complex (which is by definition a single entity) can contain two or more independent (orthogonal) modes or subshapes.

Just like the shape of a quale can change depending on whether an element is active or not, the modes or subshapes generated by a complex with a given connectivity can change depending on which elements are active. Figure 15DEF shows a grid-like system of AND-gates in three different states. When no elements are firing, as in panel D (and also when all elements are firing), the complex forms a single mode. The firing of a single element, as in panel E, causes the 4 elements targeting the one that is firing to form a single main mode with Φ = 2.4 bits. The system as a whole forms a much weaker mode, with Φ = 0.23 bits. Finally, panel F shows a more complex firing pattern that generates two overlapping modes (cyan and orange) of approximately equal entanglement, as well as additional modes (not shown) with substantial overlap with the orange and cyan modes.

#### Some phenomenological parallels: modalities and submodalities

Experience seems to divide naturally into modalities, like the classic senses of sight, hearing, touch, smell, and taste (and several others), as well as submodalities, like visual color and visual shape. What do these broad distinctions correspond to in Q? According to the IIT, modalities are sets of densely tangled q-arrows (modes) that form distinct sub-shapes in the quale; submodalities are subsets of even more densely tangled q-arrows (sub-modes) within a larger mode, thus forming distinct sub-sub-shapes. As schematically represented in Figure 15G, if the entire quale is like a very large and complex shape, modalities are like main subdivisions of its shape into sub-shapes of higher density, and submodalities are sub-sub-shapes nested within modalities, of even higher density.

In a system such as the brain, two main modes might correspond for example to the visual and auditory modalities. As would be expected, the visual and auditory system, especially early in the cortical hierarchy, are heavily interconnected within each system, and much less between systems. As illustrated schematically in Fig. 15B, such an arrangement may give rise to a large complex giving rise to a weakly tangled mode, subdivided into two main submodes. Such a complex could give rise to a quale corresponding, for example, to the simultaneous experience, by the same subject, of a bright flash and a loud bang. In other words, although the concepts “flash” and “bang” are distinct (two separate strong modes), they both fall under a single experience – a flash and a bang (the large, weaker mode). To the extent that, say by repeated exposure, a new concept were formed that strongly entangles the corresponding q-arrows, the experience would change into that of a “flashbang” or thunderbolt.

#### Some experiences appear to be “elementary,” in that they cannot be further decomposed

Sub-modes that do not contain any more densely tangled sub-sub-modes are elementary modes (i.e., elementary shapes that cannot be further decomposed). According to the IIT, such elementary modes correspond to aspects of experience that cannot be further analyzed, meaning that no further phenomenological structure is recognizable. The term qualia (in a narrow sense) is often used to refer to such elementary experiences, such as a pure color like red, or a pain, or an itch (Fig. 15G).

#### Some experiences are homogeneous and others are composite

In the Introduction we mentioned the experience of pure darkness as a paradigmatic one. Like an experience of pure light, pure red, pure blue, it shares the property of being extremely simple to describe in words: after we say that we see pure darkness, pure light, pure red, pure blue and so on, there seems to be nothing that we have left out. The corresponding quale, or shape in qualia space, is certainly not simple, as it entails presumably a large complex of informational relationships, and seeing pure darkness effectively rules out a very large number of states from the potential repertoire. In fact, the seeming “simplicity” of such pure, vivid sensations may be the main reason why the gap between neural activity and experience seems impossible to bridge. On the other hand, consider the experience of being immersed in the flow of people and traffic in a busy market street. Such an experience appears to be composed of a multitude of modalities, submodalities, and different parts, and it is very hard to describe - it may take a novelist several pages to do it justice. Though every experience is one, homogeneous experiences would be expected to translate in Q into a single homogeneous shape, and composite ones into a composite shape with many distinguishable sub-shapes (modes and sub-modes). Such a contrast is shown, in the simplest possible terms, in Fig. 15 A vs. C.

### Phenomenology and geometry: classifying and comparing shapes

If an experience is a shape in Q, in principle it should be possible to classify different experiences, or different aspects of the same experience, as one would classify shapes. Moreover, it should be possible to compare experiences or aspects thereof the way one might compare shapes, and obtain some objective indication of how similar they are. At present, a comprehensive approach to classifying and comparing qualia geometrically is not feasible, not only because of the obstacles to specifying qualia generated by realistic systems, but also because mathematical tools for comparing different qualia have yet to be developed. To provide an indication of how a geometry of phenomenology might proceed, we offer two simple examples. Thus, we suggest that topographic/categorical experiences may be organized like multidimensional grids/pyramids in Q; and that hierarchically organized experiences may be tangled both “horizontally” and vertically” into hierarchically organized subshapes in Q. Finally, we argue that treating experiences as shapes suggests a principled way of assessing their similarity and dissimilarity.

#### Of grids and pyramids, or whether aspects of experience may be classified geometrically

We recognize intuitively that the way we perceive taste, smell, and maybe color, is organized phenomenologically in a “categorical” manner, quite different from, say, the “topographical” manner in which we perceive space in vision, audition, or touch. According to the IIT, these hard to articulate phenomenological differences correspond to different basic sub-shapes in Q, such as grid-like structures and pyramid-like structures. In turn, these emerge naturally from the underlying neuroanatomy and neuronal activity patterns.

**Figure 16:**
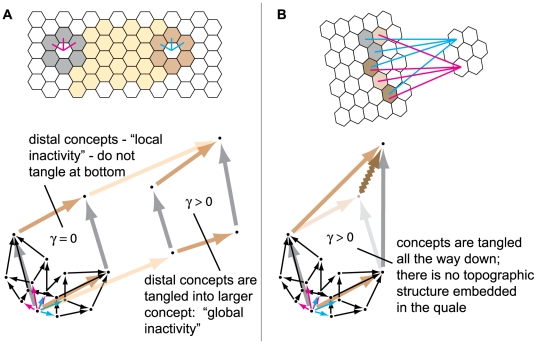
The qualia generated by topographical grids and categorizing pyramids. (A) A honeycomb grid and a schematic representation of part of the quale generated by the grid. In the grid, each element is bidirectionally connected to its 6 neighbors, and fires if it receives 3 or more spikes. The cell at the center of the gray area is silent, and so generates the concept “local activity below threshold”. Three of the connections targeting the cell are shown in pink; the corresponding q-arrows at the bottom of the quale are tangled into the overarching concept given by the larger gray q-arrow. Similarly for the cell at the center of the brown area that – as shown in the quale – tangles the connections shown in blue. The quale shows how the grid generates two concepts for “local activity below threshold” in two different regions (the two deformed cubes generated by pink and cyan q-arrows). The concept generated by th pink q-arrows taken as a whole is represented by a gray q-arrow at the bottom of the quale; similarly a brown q-arrow is drawn for the concept generated by the cyan q-arrows as a whole. The combined concept “activity below threshold in the gray and brown regions” *does not exist for the grid* because the brown and gray q-arrows are *not* tangled at the bottom of the quale. The overarching informational relationship generated by the gray, beige and brown areas together does form a single concept in the quale “regional activity below threshold”. (B): Part of a categorizing pyramid extracting invariants from a grid and a schematic of the quale. The categorizing pyramid has near all-to-all connectivity, so there is no topographic structure, which is reflected in the quale by tangling “all the way down”. In contrast to the grid, where the topographic structure serves to prevent concepts from tangling at the bottom of the quale, giving the experience a spatial aspect, the all-to-all connectivity results in all concepts tangling into a single indivisible experience similar to color or smell.

Many sensory areas, especially early on the cortical hierarchy, are organized topographically [34], very much like a grid. What does this basic neuroanatomical arrangement contribute to the quality of experience? In other words, what it is like to be a grid? Figure 16A shows a honeycomb grid: elements receive connections from 6 neighboring elements and fire if they receive more than 3 spikes. Consider a silent element on the left, surrounded by 6 gray elements, with 3 out of its 6 afferent connections shown in pink. The concept generated by its afferent connections can be characterized as “local activity below threshold.” In Q, the corresponding q-arrow is the gray one that tangles the pink-q-arrows at the bottom of the quale (generated by the pink connections). Consider next another silent element on the right (surrounded by brown elements, with afferent connections shown in blue), which is spatially removed from the first, and which generates another instantiation of the concept “local activity below threshold.” In Q, the corresponding concept is a brown q-arrow that tangles the blue q-arrows at the bottom of the quale. The two concepts (gray and brown q-arrows) are not tangled at the bottom of the quale, so there is no concept corresponding to “local activity below threshold in these two separate areas.”

However, if the two elements are neighbors, the concept generated by their connections tangle, since afferents that are topographically adjacent jointly specify an actual repertoire more precisely than if considered independently. The resulting tangled q-arrow, shown in beige, corresponds to the concept “larger patch of local activity below threshold.” In this manner, one neighboring element after the other, entanglement progressively expands q-arrows in a topographically continuous manner, until at the top of the quale all q-arrows (here the gray, brown and beige ones) become tangled into the concept “activity below threshold everywhere.“In the end, the geometry of the quale would reflect the nearest neighbor architecture of the grid, building concepts from local pieces centered on elements, up to a single global gestalt (the grid forms a single mode). An element firing would then warp the tangled shape generated by the silent grid corresponding to the concept “local activity above threshold here and below threshold everywhere else.” In this vein, the example in Fig. 16A could be interpreted as a cartoon model of the spatial aspects of vision, audition, or somesthesia.

Consider now a simple system that is organized like a categorizing pyramid (Figure 16B). Here, each element in the upper level, through afferents originating throughout the lower level, generates a concept that globally categorizes its input, along the lines of Fig. 13. As in Fig. 13, each concept is assumed to be tangled in Q, meaning that the sum of the information generated by all afferents is more than the information generated by the afferents separately. Moreover, as in Fig. 13, each concept is assumed to specify a different set of firing patterns at the lower level, that is, each concept is different, and different concepts are tangled, so that together they generate more information than separately. In Q, afferents of different elements are tangled starting already at the bottom of the quale and all the way up to the top. In contrast to the grid, where the topographic structure prevents concepts generated by distant elements from tangling at the bottom of the quale, thereby giving the experience a “spatial” aspect, the forward all-to-all connectivity of the pyramid in the cartoon model of Fig. 16C,D results in all concepts tangling from the beginning, perhaps similar to color, taste or smell.

This example is also meant to illustrate how basic features of neuroanatomical organization contribute to determining the quality of experience. On one hand, there is overwhelming evidence that different brain areas contribute different aspects to the quality of consciousness. On the other hand, the present approach suggests that the contribution of different neuroanatomical structures to experience is not direct (and mysterious). Instead, the contribution of different brain areas to experience would be mediated (and explained) by how their connectivity, together with their activity patterns, specifies shapes in qualia space.

#### Phenomenological hierarchies: building shapes vertically and horizontally

Much of experience is hierarchically organized [35],[36] and, perhaps not coincidentally, so is the organization of sensory pathways in the cortex [37]–[39]. Take seeing a face: we see at once that as a whole it is somebody's face, but we also see that it has parts such as hair, eyes, nose and mouth, and that those are made in turn of specifically oriented segments. Correspondingly, neurophysiologic experiments indicate that neurons in early visual areas respond to oriented segments. Presumably, there are also neurons responding to eyes, noses and mouths. In areas higher in the visual hierarchy, there are neurons that respond to faces, often in a position invariant manner. How can the informational relationships generated by these neurons and their mechanisms combined to give rise to the percept of a face?

**Figure 17:**
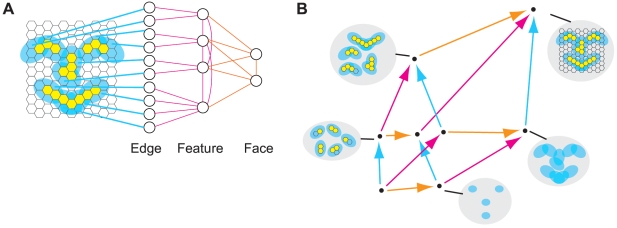
Hierarchical experiences. (A): Higher-order feature detectors extract a hierarchy of patterns (edges, features, and faces) from a retina-like grid. (B): A schematic depiction of the quale generated by the hierarchy; since each pattern-detector contains many elements and connections, the actual quale will be vastly more complicated than the simple cartoon shown here. The actual repertoires generated along two q-edges are shown. First, consider the clockwise q-edge. The cyan connections – targeting the edge detectors – specify that the image presented to the retina contains certain edges. The edge and feature detectors taken together specify that the edges coalesce into features such as a mouth, nose and eyes. Finally, all the connections in the hierarchy specify the particular face that is shown to the retina. Going around anti-clockwise, the “face” connections on their own specify that the retina-grid was presented with a face-like object, however, the details of the face are unspecified, since the concepts for mouth etc. are not generated by the face-neurons. Engaging the connections targeting the feature-neurons fills out some of the details of the face, the broad outlines of how the nose, mouth and eyes appear. Finally, adding connections targeting the edge-neurons specifies the face precisely. The informational relationships generated by neurons in a tangled quale cannot be described in isolation.

Consider the diagram in Fig. 17A. Feature detectors in a primary cortical area specify that there may be some edges in some locations of the retina grid. Tangled “horizontally” in a topographic manner, meaning with connections afferent to other neurons in the same area, they specify a certain contour. In Q, as illustrated schematically in Fig. 17B (clockwise q-edge of the quale), this contour information provides a natural *context* on top of which to tangle, “vertically,” the contribution of neurons in a higher area whose connections specify the presence of eyes, nose, and mouth. On top of this richer context, “face” neurons in even higher areas are tangled, again vertically, to specify a face.

The counter-clockwise edge of the quale illustrates how the “face” connections on their own specify that the retina-grid was presented with a face-like object, ruling out alternatives such as house-like, car-like, and so on. However, the details of the face are missing, and the face-neurons cannot specify how the face looks. Engaging the connections targeting the feature-neurons fills out some of the details of the face, the broad outlines of nose, mouth and eyes, and more details are added by the edge-neurons. In the context provided by the edge and feature neurons, the face-neurons again rule out the alternatives, however now the alternatives are far more detailed. The q-arrows specified by these various sets of connections would be expected to be highly tangled, embodying relationships within and across levels, and generating more information that the sum of their component q-arrows. For example, informational relationships constituting a “face” would be more densely tangled than unnatural combinations such as one eye and a lower lip. Back-connections and lateral connections may play a role (beyond their role in learning and attention) by allowing higher-order invariants to inform – and so tangle with – lower order invariants. Indeed, psychophysical experiments have shown that feature recognition involves extensive filling-in of lower-order features [40].

Altogether, according to the present approach, the experience of a person's face, with its faceness, its eyes, nose and mouth, its precise contour and spatial location, would not be captured by any individual neuron or population of neurons, whether face cells or not, whether firing or not, whether synchronous or not, but by the generation of a set of informational relationships within a complex, i.e. a particular sub-shape in Q.

#### Phenomenological similarities and dissimilarities: comparing shapes

Some experiences are more alike than others. Blue is certainly different from red (and irreducible to red), but clearly it seems even more different from middle C on the oboe. In the IIT framework, colors correspond to different sub-shapes of the same kind (say pyramids pointing in different directions) and sounds to very different sub-shapes in Q. In principle, such subjective similarities and differences can be investigated by employing objective measures of similarity between shapes [41]–[43]. For example, one could consider the number and kinds of symmetries involved in specifying shapes that are generated in Q by different neuroanatomical circuits. Though this perspective will not be pursued here, in principle it opens the door to mathematical approaches already employed in other fields or susceptible to theoretical development.

Considering the quantity of consciousness as given by the repertoire of states that can be discriminated by a single system, and its quality by the shape of the set of informational relationships generated by its connections, may also shed some light on the effects of splitting the brain along the corpus callosum in severely epileptic patients [2]. Such patients appear to possess two distinct consciousnesses, one localized in each hemisphere. Particularly surprising is that the dominant (verbal) hemisphere appears to behave similarly to an intact brain, and reports largely similar experiences [44]. As shown in Section 7 of Text S1, the shape of the quale generated by certain systems can be indifferent to the number of elements if the system contains redundancies or degeneracies [45]. Therefore, it is possible that the quale generated by a single hemisphere may be similar, in a quantifiable sense, to the quale generated by the entire brain, entailing comparable quantity and quality of consciousness.

### Seeing red

In this last section, we revisit the question of the quality of consciousness by considering a paradigmatic quale – say seeing red – and discussing how such an experience should be thought of, at least in principle, from the point of view of the IIT. We choose a color not only because it is a traditional example in philosophy, but because we can lend it a minimum of concreteness by referring to some evidence from neurology and neuropsychology (another clinical syndrome that would lend itself naturally to this sort of analysis is neglect [46]). This final demonstration is inevitably bare-bones. Nevertheless, it should serve the purpose of illustrating how, according to the IIT, the “redness” of red, and similarly any qualitative aspect of experience, is not specified by the firing of particular neurons, nor by particular patterns of activity or correlations, nor is it a property of certain anatomical circuits, but it exists only at the level of the set of informational relationships generated by a complex of elements in a certain state. Specifically, the “redness” of red, and similarly any qualitative aspect of experience, corresponds to a specific q-fold within a quale, generated by the activation of a set of specialized mechanisms. As such, it exists only in the context of the quale, just like a particular convexity in a complex solid only exists in the context of the solid. This perspective also implies that specific qualities of consciousness, while generated by a local mechanism, cannot be reduced to it.

#### The NCC of red specifies the “redness” of red only in the full context of a quale

Consider, then, the experience of seeing a pure color, such as red. The evidence suggests that the “neural correlate” or NCC [47] of color, including red, is probably a set of neurons and connections in the fusiform gyrus, maybe in area V8. Ideally, neurons in this area are activated whenever a subject sees red and not otherwise, if stimulated trigger the experience of red, and if lesioned abolish the capacity to see red. Certain subjects with dysfunctions in this general area, who are otherwise perfectly conscious, seem to lack the feeling of what it is like to see color, its “coloredness,” including the “redness” of red. Such achromatopsic subjects cannot experience, imagine, remember and even dream of color, though they may talk about it, just as we could talk about echolocation, from a third person perspective [48]. Contrast such subjects with vegetative patients, who are for all intents and purposes unconscious. Some of these patients may show behavioral and neurophysiologic evidence for residual function in an isolated brain area [49]. Yet it seems highly unlikely that a vegetative patient with residual activity exclusively in V8 should enjoy the vivid perceptions of color just as we do, while being otherwise unconscious.

The IIT provides a straightforward account for this difference. To see how, consider again Fig. 9: call r the connections targeting the “red” neurons in V8 that confer them their selectivity, and non-r (¬r) all the other connections within the main corticothalamic complex. Adding r in isolation at the bottom of Q (null context), yields a small q-arrow (called the *down-set of red* or ↓r) that points in a direction representing how r by itself shapes the maximum entropy distribution into an actual repertoire. Schematically, this situation resembles that of a vegetative patient with V8 and its afferents intact but the rest of the corticothalamic system destroyed. The shape of the experience or quale reduces to this q-arrow, so its quantity is minimal (Φ for this q-arrow is obviously low) and its quality minimally specified: as we have seen with the photodiode, r by itself cannot specify whether the experience is a color rather than something else, such as a shape, whether it is visual or not, sensory or not, and so on.

By contrast, subtract r from the set of all connections, so one is left with ¬r. This “lesion” collapses all q-arrows generated by r starting from any *context*, that is, it folds the quale along the *q-fold* specified by r, as we saw in Fig. 10. Prominent within the q-fold generated by r in the quale is the informational relationship that starts from the full context, provided by all other connections ¬r, and reaches the top of the quale, called the *up-set of non-red* (↑¬r). This q-arrow will typically be much longer and point in a different direction than the q-arrow generated by r in the null context at the bottom of the quale, as we saw in Fig. 9. This is because, the fuller the context, the more r can shape the actual repertoire. Schematically, removing the q-fold of r resembles the situation of an achromatopsic patient with a selective lesion of V8: the bulk of the experience or quale remains intact (Φ remains high), but a noticeable feature of its shape, the q-fold specified by r, collapses. According to the IIT, it is this q-fold that constitutes the “redness of red.” More precisely, the feature of the shape of the quale specified by the up-set of non-red, which includes as a context all other connections, including those specifying other colors, captures the quality or “redness” with respect to other colors. Lower q-arrows in the q-fold of red contribute to specifying the “coloredness” of red with respect to other visual attributes, such as shape or motion, lower ones its “visualness” with respect to other sensory modalities, its “perceptualness” as opposed to thought, and so on.

It is worth remarking that, while the quality of red specified by the q-fold of r in the above example refers to one particular experience, it is in principle conceivable to determine, in an objective manner, what different experiences described as red by a conscious subject, or even by different subjects, may have in common. Once again, one would need to establish what aspects of the shape of different qualia remain similar across different experiences of red from the same subject or different subjects.

The last example also shows why specific qualities of consciousness, such as *the “redness” of red, while generated by a local mechanism, cannot be reduced to it*. If an achromatopsic subject without the r connections lacks precisely the “redness” of red, whereas a vegetative patient with just the r connections is essentially unconscious, then the redness of red cannot map directly to the mechanism implemented by the r connections. However, the redness of red can map nicely onto the informational relationships specified by r, as these change dramatically between the null context (vegetative patient) and the full context (achromatopsic subject).

### Summary and conclusions

In this paper, we have briefly reviewed the notion of integrated information, the amount of information generated by a complex of elements above and beyond the information generated by its minimal parts, measured by Φ. We have then introduced the notion of qualia space (Q) as a space with an axis for each possible state of the complex. Each submechanism of the complex specifies a probability distribution of system states, corresponding to a point in Q. Arrows between points (probability distributions) in Q (q-arrows) define informational relationships among the elements of the complex (the effective information matrix [1]). Together, all these informational relationships specify a quale Q(mech, x_1_), which is a shape (high dimensional solid or polytope) in Q space. We argued that this shape completely and univocally characterizes the quality of a conscious experience. Φ – the height of this solid – is the quantity of consciousness associated with the experience. High Φ allows “breathing room” for the informational relationships within a complex to express themselves, while if Φ is reduced the quale collapses.

We have examined several corollaries that can be derived from these premises. For example, only informational relationships within a complex are part of the same quale. The shape of the quale is always determined by the mechanism (connectivity) and the state of the elements (activity pattern) considered together. Thus, two systems having exactly the same activity pattern may give rise to completely different qualia, depending on their mechanism. In the limit of no mechanism – a system that merely copies its state from another one – no quale is generated. Conversely, exactly the same quale may be generated by two systems that differ both in terms of connectivity and activity patterns. For example, a silent AND-gate and a firing OR-gate generate isomorphic qualia. On the other hand, in more complex systems many symmetries are likely to be broken, making it extremely unlikely that two different systems that are sufficiently complex may generate the same quale.

Some of the results derived from the present approach lend themselves to a neurophysiologic interpretation. For example, we have seen that both active and inactive elements specify a quale. Thus, a system in which all elements are silent can still specify a quale with a complex shape. On the other hand, while elements that are inactive contribute to specifying a quale, elements that are inactivated (incapable of becoming active) do not, even though, from an extrinsic perspective, the pattern of activity may not have changed. Also, when an element within a complex becomes active, it changes the shape of the quale. The implication is that the meaning of the firing of a given element (neuron) is given not by what its extrinsically imposed label might be (a “red” neuron or a “face” cell), nor by the information it broadcasts to other elements (‘red” or “face”), but by the new shape in Q it contributes to specifying.

Generating more concrete links between the techniques developed here and experimental data requires robust causal models of neuronal activity. Recently, a large body of work on dynamic causal modeling (DCM) has been developed attacking exactly this problem, for example [50]–[52]. DCM takes a perturbational approach to modeling neuronal interactions, treating an experiment as a targeted perturbation of a set of interacting neuronal populations. Causal models are fitted to experimental data using Bayesian techniques; thus, the output of DCM – a causal model – is exactly what the IIT requires as an input. Connecting the two formalisms will require some effort since DCM uses continuous rather than discrete models, however this appears to be a technical rather than conceptual obstacle (see Section 1 of Text S1). DCM thus provides a possible bridge between empirical data and the approach developed in this paper.

It should be pointed out that this paper investigates the quality of the informational relationships generated by a system without any reference to the environment and to the issues posed by sensory processing or by learning. The important problem of how a system can integrate information in such a way as to match its environment will be treated in future work.

The present approach can help to rephrase basic phenomenological and neuropsychological observations in a geometrical language. For example, we have seen how entanglement – which occurs when a submechanism gives rise to an informational relationship that cannot be decomposed into its component relationships, generates concepts and modes Entanglement is necessary for dimensionality reduction from many inputs to a single output, which is an essential informational requirement for neurons. Entanglement helps to increase integrated information, ensuring that a complex can make highly informative discriminations as a single entity.

We have also seen how informational relationships can be refined through learning, thereby generating a more differentiated experience or quale. After introducing the notion of modes – sets of densely tangled informational relationship, we have argued that the subdivision of experience into modalities and submodalities corresponds to subshapes (modes) in Q; that qualia in the narrow sense are elementary modes (not further decomposable such as the “redness” of red); and that homogeneous/composite experiences are homogeneous/composite shapes. Also, the notion of modes clarifies how some phenomenological aspects may appear largely orthogonal (say visual and auditory details) and yet be part of the same experience or quale in the broad sense.

Finally, we have argued that it may in principle be possible to obtain a geometrical classification of aspects of experience in terms of the shape in Q of the underlying informational relationships. As a tentative example, we suggested that topographic/categorical experiences may be like grids/pyramids in Q; and that hierarchically organized experiences are tangled both “horizontally” and vertically” into hierarchically organized subshapes. We have also argued that the similarity between experiences reduces to similarities between shapes. Finally, we have argued that specific qualities of consciousness, such as the “redness” of red, while generated by a local mechanism, cannot be reduced to it, but require considering the shape of the entire quale, within which they constitute a q-fold.

These abstract geometrical notions may seem at first to be far removed from the immediacy of experience. At present, due to the combinatorial problems posed by deriving the shape of the quale produced by systems of just a few elements, and to the additional difficulties posed by representing such high-dimensional objects, the best one can hope for is to show that the language of Q can capture, in principle, some of the basic distinctions that can be made in our own phenomenology. Ultimately, however, the goal of the present framework is to offer a principled way to begin translating the seemingly ineffable qualitative properties of experience into the language of mathematics.

## Supporting Information

Text S1 Supplementary information
(1.40 MB PDF)
