# Supplementary material for: Qualia: The Geometry of Integrated Information
Source: PLoS Comput Biol. 2009 Aug 14;5(8):e1000462. doi: 10.1371/journal.pcbi.1000462 (PMC2713405; doi:10.1371/journal.pcbi.1000462)
Supplement: Text S1 — Supplementary information (1.40 MB PDF) [file pcbi.1000462.s001.pdf]

Qualia: The Geometry of Integrated Information

David Balduzzi - balduzzi@wisc.edu; Giulio Tononi - gtononi@wisc.edu

Supplementary Information

The main text makes use of two thought experiments – involving a photodiode and a digital camera – to argue that the quantity of consciousness experience can be measured as the integrated information generated by a system; and further that the quality of an experience is captured by the shape of the set of informational relationships generated by the system. The supplementary information complements the main text with a more technical discussion.

SI-1 Effective information

The photodiode thought experiment demonstrates that different systems respond differently to different stimuli. From this we deduce that systems generate information about their prior state by discriminating between (responding differently to) possible inputs.

First, for reference, we recall the formula for relative entropy [1]. Given two probability distributions p and q, the entropy of p relative to q is:

$$H\big[p \,\|\, q\big] = \sum_i p_i \log_2 \frac{p_i}{q_i}.$$

Motivation for relative entropy as a measure of information is provided in [2]. It can be shown that relative entropy is always nonnegative $H\big[p \,\|\, q\big] \geq 0$ for all p and q, and that $H\big[p \,\|\, q\big] = 0$ if and only if $p = q$.

SI-1.1 Shannon information is expected surprise

Suppose we have a random variable X with probability mass function $p(x_i) = Pr\{X = x_i\}$. Shannon information, or Shannon entropy, of X is $I(X) = -\sum_i p(x_i)\log_2 p(x_i)$. We interpret it as follows [1]. Before the event, the probability of various outcomes is given by the prior distribution: the probability mass function $p(x_i)$. After event $\{X = x_k\}$, the probability of various outcomes is given by the posterior distribution δ_k (the Kronecker delta function):

$$\delta_k(x_i) = Pr\{X = x_i | X = x_k\} = \begin{cases} 1 & \text{if } i = k \\ 0 & \text{else.} \end{cases}$$

The information arising from event $\{X = x_k\}$ is the surprise (or self-information) $I(x_k) = -\log_2 p(x_k)$. Rewrite surprise as the entropy of the posterior distribution relative to the prior distribution:

$$I(x_k) = H\Big[\text{posterior} \,\Big\|\, \text{prior}\Big] = H\Big[\delta_k \,\Big\|\, p\Big] = \sum_i \delta_k(x_i) \log_2 \frac{\delta_k(x_i)}{p(x_i)} \tag{S1}$$

Notice that all the terms in the sum where delta function is zero vanish, leaving only the surprise term.

Surprise (self-information) results from the contrast between the posterior and prior distributions. Surprise from event $\{X = x_k\}$ is high in bits if the prior probability $p(x_k)$ is low. One way this can occur is if the random variable X represents a large set of equally likely events. In this case high surprise results from the contrast between a specific actual outcome, $\{X = x_k\}$, and the large number of possible outcomes.

Shannon information is expected surprise:

$$I(X) = \mathbb{E}\Big\{H\big[\delta_i \| p\big] \,\Big|\, p(x_i)\Big\} = \sum_i p(x_i) \cdot H\big[\delta_i \| X\big],$$

showing that Shannon information quantifies the amount of information given (alternatives ruled out) by the random variable *on average.* Shannon information is high for repertoires with minimal structure (corresponding to maximum prior ignorance or maximum entropy [3]). This is because maximum ignorance results in maximal surprise – on average. High surprise results from the contrast between an aspecific prior distribution and a specific posterior. The contrast is implicit in the formula for Shannon information, and is made explicit by unfolding Shannon information as an expectation of relative entropies (surprises).

Note that effective information is measured for a single event (such as a photodiode turning ON) rather than for a set of events, so effective information is analogous to surprise rather than Shannon information.

SI-1.2 The actual repertoire specifies the discriminations performed by a (sub)mechanism

By responding differently to different inputs, a system implicitly discriminates between stimuli. The actual repertoire makes the discriminations explicit. The system has the following properties:

1. it has a causal mechanism that responds differently to different inputs;
2. there is set of potential states that can be communicated between elements, which depends on the causal mechanism (how the system is built); and
3. that its prior state caused (led to) state x_1.

The **causal mechanism** is a transition probability matrix, $p^{\mathfrak{Conn}_X}\big(x_1|do(x_0)\big)$, which determines how the system responds to inputs. We use the $do(-)$ operator [4] to indicate that x_0 is forcibly imposed on the system through a causal intervention.

The **potential repertoire** is determined by the causal mechanism. It lists the outputs that can be communicated across the connections in the system. Given a system with a mechanism we have no *a priori* information regarding which inputs on the list are more or less likely than others, so we place the maximum entropy [3] or uniform probability distribution on potential inputs. The potential repertoire is denoted by $X_0(maxH)$.

There is no difference in principle between a system and a subsystem: a subsystem is a subset of a system. We label subsystems with subsets of the set of connections, and refer to their mechanisms as submechanisms of the system as a whole. Inputs from outside the subsystem cannot be accounted for by causal interactions within the subsystem, and so are treated as *extrinsic noise.* Suppose we have system X, with set of connections $\mathfrak{Conn}_X$. The causal submechanism $p^{\mathfrak{m}}(x_1|do(x_0))$ across connections in $\mathfrak{m} \subset \mathfrak{Conn}_X$ is computed by imposing maximum entropy noise *on the outputs of each connection in* $\neg\mathfrak{m}$ *independently*:

$$p^{\mathfrak{m}}(x_1|do(x_0)) = \sum_{n_0 \in \neg\mathfrak{m}} p^{max}(n_0) \cdot p^{\mathfrak{Conn}_X}\left(x_1|do(x_0), do(n_0)\right).$$

The **actual repertoire** explicitly describes the discriminations made by the mechanism, in choosing the current output state x_1 rather than some other state. The actual repertoire is then computed via Bayes' rule, which acts as a bookkeeping tool by explicitly keeping track of which perturbations cause (lead to) x_1 and which do not.

$$p\Big(x_0(\mathfrak{m}, x_1)\Big) = \frac{p^{\mathfrak{m}}\left(x_1|do(x_0)\right) \cdot p^{max}(x_0)}{p^{\mathfrak{m}}(x_1)}. \tag{S2}$$

We use notation $X_0(\mathfrak{m}, x_1)$ to denote the actual repertoire specified by interactions across submechanism $\mathfrak{m}$ that cause the system to enter state x_1.

The potential and actual repertoires are analogous to the prior and posterior repertoires discussed in the subsection on Shannon information. For this reason, in earlier work the potential and actual repertoires were referred to as the *a priori* and *a posteriori* repertoires respectively [5].

SI-1.3 Effective information quantifies the discriminations performed by a (sub)mechanism

Effective information is analogous to surprise rather than Shannon information since it is computed for a single event. Conceptually, the two measures differ as follows. Surprise takes a probability distribution and an event as arguments and computes, in bits, how unlikely an event was prior to its occurrence. Effective information takes a

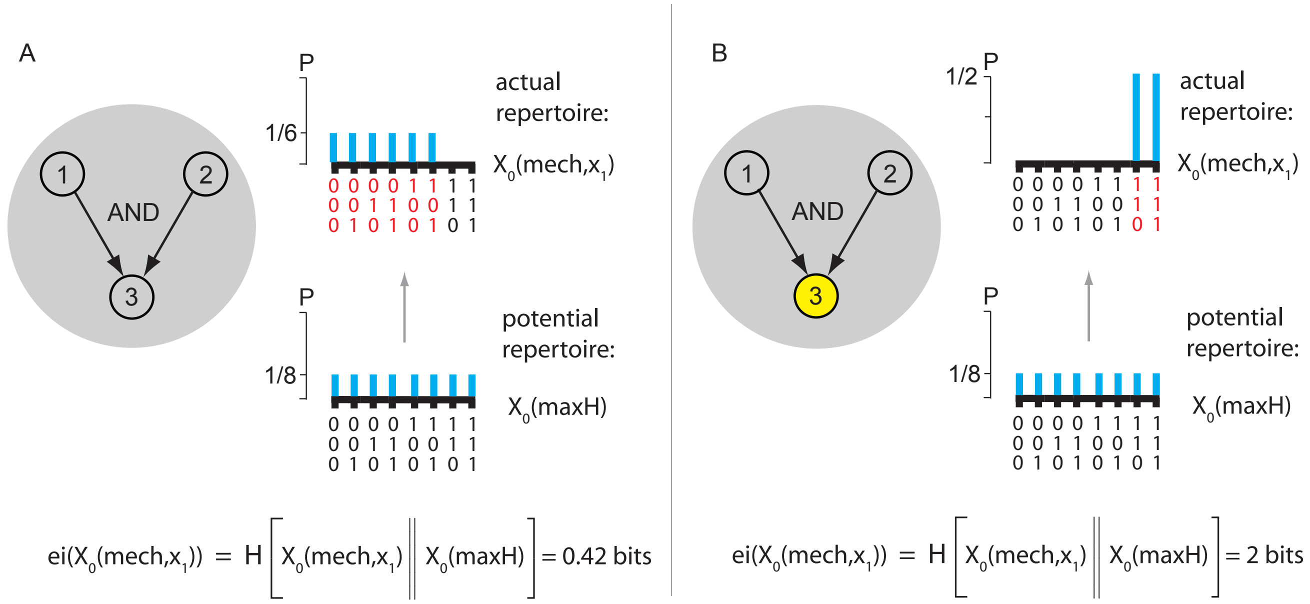

Figure S1: Effective information for an AND-gate. A system containing a single *AND*-gate. Element n^3 fires at time $t = 1$ if and only if it receives 2 spikes at $t = 0$. Elements n^1 and n^2 are set externally; their responses cannot be accounted for by causal interactions within the system. (AB): The potential and actual repertoires specified by a silent and firing *AND*-gate respectively, along with the effective information generated.

system (a mechanism) and a state as arguments and computes how the mechanism discriminates between potential prior states by choosing the current state. Thus, surprise is measured from the perspective of a 3rd party who is given the prior distribution $p(x_i)$ and observes the occurrence of event $\{X = x_k\}$; whereas effective information takes the intrinsic perspective of the mechanism.

How much information does a system generate when it responds to a perturbation by choosing a state? Effective information generated by a submechanism is the entropy of the actual repertoire relative to the potential repertoire:

$$ei\Big(X_0(maxH) \to X_0(\mathfrak{m}, x_1)\Big) = H\Big[X_0(\mathfrak{m}, x_1)\,\Big\|\,X_0(maxH)\Big]. \qquad \text{(S3)}$$

Effective information is modeled on surprise, Eq (S1), and computed as

$$ei\Big(\text{potential} \to \text{actual}\Big) = H\Big[\text{actual}\,\Big\|\,\text{potential}\Big]$$

or

$$ei\Big(\text{actual}(submech) \to \text{actual}(mech)\Big) = H\Big[\text{actual}(mech)\,\Big\|\,\text{actual}(submech)\Big].$$

Effective information quantifies how informative the discriminations performed by *mech* are in the context provided by *submech*. It thus takes the intrinsic perspective on the discriminations – the information-theoretic 'work' – performed by the mechanism.

In both surprise and effective information there is a particular outcome: event $\{X = x_k\}$ and state x_1 respectively. Surprise measures the *a priori* likelihood of the event: if the event was judged unlikely before it happened, then it is surprising. Effective information measures how the mechanism discriminated between states by choosing x_1. If many prior states cause x_1 then the link is weak and effective information is low; if few prior states out of a large potential repertoire cause x_1, then x_1 is the result of a highly informative discrimination by the mechanism. Notice that: *(i)* surprise depends on the choice of *a priori* probability distribution and *(ii)* does not take into account the mechanism of the system. Thus, a system that fires randomly with 50% probability and a "photodiode" that fires iff it receives a spike both generate 1 bit of surprise, but only the photodiode generates effective information.

Fig. S1 shows an example system containing an *AND*-gate. The potential repertoire in the system of 3 binary

elements contains $2^3 = 8$ perturbations, $000 \ldots 111$, all of which are weighted equally. The actual repertoires specify how the mechanism of the system discriminates between perturbations. When the *AND*-gate is silent, it specifies that the prior state of elements n^1 and n^2 was one of $00, 01, 10$. The prior state of element n^3 has no causal effect on the system, and so is lost, which can also be read off the actual repertoire. Similarly, when the *AND*-gate is firing, it specifies that the prior state of $n^1 n^2 = 11$, and nothing is known about the prior state of n^3. Effective information is 0.42 bits for the silent *AND*-gate (ruling out 2 of 8 potential perturbations) and 2 bits for the firing *AND*-gate (ruling out 6 of 8).

The submechanism of the system given by connection c^{13}, is computed by treating the other connection c^{23} as extrinsic noise. In this case, the transition probability matrix is

$$p^{\mathfrak{m}}\big(n_1^3 | do(n_0^1)\big) = \left\{ \begin{array}{c|cc} & n_1^3 = 0 & n_1^3 = 1 \\ \hline n_0^1 = 0 & 1.0 & 0.0 \\ n_0^1 = 1 & 0.5 & 0.5 \end{array} \right.$$

SI-1.4 The intrinsic perspective

This section briefly explains what we mean by the intrinsic versus extrinsic perspectives.

The **intrinsic perspective** is the point of view of the system itself, given its current state and causal mechanism. At any given time, any physical system is in a certain state and is endowed with a certain causal mechanism; these are intrinsic, observer independent properties. According to the IIT, purely by virtue of being in that state and having that mechanism, the system reduces uncertainty ("integrated information" is generated) about which of its possible states might have caused its present state, and which might not. This "intrinsic" information captures "the differences that make a difference" to the system itself [6].

By contrast, the **extrinsic perspective** is that of an observer external to the system, given the system's state and the observer's expectations. Upon observing a given state, uncertainty is reduced ("surprise" is generated) with respect to some prior distribution that captures the expectations of the external observer. In general, this prior distribution is based on observing and recording system inputs and outputs over extended periods of time, while the underlying causal mechanism is ignored. Taking the extrinsic perspective, one typically asks questions about how "extrinsic" information (i.e. average surprise) is encoded, communicated or stored.

SI-1.5 Measurements take the perspective of the measuring apparatus

A state can be interpreted as the result of a measurement a system performs on itself. Each measurement specifies an actual repertoire, and the information generated by the measurement is the entropy of the actual repertoire relative to prior ignorance. We briefly relate the theory to the language of measurement in classical mechanics.

Suppose, for example, that we wish to measure kinetic energy in system X with state space $\mathcal{S}(X)$. Formally, kinetic energy is given by a function

$$f_E : \mathcal{S}(X) \to \mathbb{R}$$

mapping states to energy levels. The function f_E is an abstract representation of the action of a measuring apparatus on the system, which outputs something interpreted as a real number. Suppose we take a measurement with result $r \in \mathbb{R}$. Assuming maximal prior ignorance, all we know about the system is that its state, at the time the measurement was performed, was in subset $f_E^{-1}(r)$ of state space. Moreover, each of these states is equally likely. The measurement, function f_E and state r, thus specifies an actual repertoire. The information generated by the measurement is quantified by computing the entropy of the actual repertoire relative to the maximum entropy distribution on $\mathcal{S}(X)$. If there are few states with energy r the measurement is informative; conversely, if most states have energy r the measurement is not.

Any measurement is the outcome y_1, of interactions across a measuring apparatus (submechanism) $\mathfrak{m}$ that generates information about the possible prior states of a system X_0. Extrinsic noise is reduced as much as possible in the design of the experiment, and the remainder is either ignored or averaged over. The experimenter takes the perspective of the measuring apparatus $\mathfrak{m}$ and uses outcome y_1 to make inferences about X_0.

More precisely, given a set $\mathfrak{m}$ of connections, we have corresponding mechanism $p^{\mathfrak{m}}\big(y_1 | do(x_0)\big)$. The transition

probability matrix induces a linear operator (modulo normalization) on $\mathcal{Q}(X)$:

$$A_{\mathfrak{m}} : q(x_0) \mapsto \left[\frac{p^{\mathfrak{m}}(y_1|do(x_0))}{p(y_1)}\right] \cdot q(x_0). \tag{S4}$$

Operator $A_{\mathfrak{m}}$ specifies the result of measurement $\mathfrak{m}$ on prior uncertainty q. In particular $A_{\mathfrak{m}}\Big[X_0(maxH)\Big] = X_0(\mathfrak{m}, y_1)$.

According to the IIT there is something that it is like to be an integrated system: to make discriminations firsthand and as a single entity. A related point of view has been advocated in [7, 8], where it is pointed out that many of the paradoxes arising in quantum mechanics follow from the assumption that there is an observer independent ("objective") reality. Rovelli hypothesizes that "quantum mechanics is a theory about the physical description of physical systems relative to other systems, and this is a complete description of the world". Thus, any observation requires an observer, where each observer is itself a physical system. This is compatible with our position that information is generated by a mechanism in a particular state, and does not exist in the abstract. We introduce a sharp distinction between the past ($t = 0$) and the present ($t = 1$), and claim that the system in its present state generates information about the prior state by performing a measurement: ruling out some alternatives and specifying others in the form of an actual repertoire. One advantage of introducing this distinction is that it is possible to quantify how much information a system generates about itself as a whole (the total partition), rather than necessarily partitioning systems into an observing and an observed part. However, according to the IIT, information only exists if it is integrated information; that is, in so far as it is generated by a single entity (a complex).

SI-1.6 Generalizing the set of outputs

The examples in the paper consist of binary elements (with outputs 0 and 1). This is purely for expository convenience; the framework generalizes naturally to arbitrary sets of outputs as follows. Consider a system X containing elements $n^1, \ldots, n^k$. Suppose element n^j has set of outputs $\mathcal{O}_j$. In the paper, we always have $\mathcal{O}_j = \{0, 1\}$. The state space of the system is the product $\mathcal{S}(X) := \prod_{j=1}^{k} \mathcal{O}_j$ and qualia space is the set of all functions from $\mathcal{S}(X)$ to $\mathbb{R}$

$$Q(X) := \{f : \mathcal{S}(X) \to \mathbb{R}\}.$$

In the case $\mathcal{O}_j = \{0, 1\}$ for all j, we recover the qualia space used in examples in the paper.

Continuous variables pose technical challenges. However, many of these have already been dealt with in other contexts. For example, suppose we have a system where each element can produce outputs in the interval $\mathcal{O} = [0, 1]$, as in the case of certain sigmoid-neurons. In this case $\mathcal{S}(X) = [0, 1]^k \subset \mathbb{R}^k$. Qualia space is then the function space

$$Q(X) = \mathcal{F}([0, 1]^k) = \{f : [0, 1]^k \to \mathbb{R} \,|\, f \text{ satisfies an integrability condition } \}.$$

Although this space is infinite dimensional, it has been intensively studied and is well understood [9,10]. Extending the formalism to smoothly varying submechanisms will require working with continuous lattices [11]. Relative entropy is well-behaved in a continuous setting (see [1] and SI-11 of [5]) suggesting that the theory can be extended to continuous variables. In fact, it is often easier to work with continuous objects than discrete objects since there is more structure at hand, and analytic methods can be applied instead of combinatorics.

SI-1.7 Repertoires can be interpreted as random variables

We explain how the quale can be understood in terms of random variables.

A system X is in state x_1. An event has occurred: the system's mechanism has discriminated between potential inputs at time $t = 0$ in choosing state x_1 rather than some other state (which it may have done had it received a different input at $t = 0$). What is the set of all possible events? The set of all possible discriminations between potential states, i.e. the set of all subsets (powerset) of the state space $\mathcal{S}(X)$. We denote the powerset, which is a σ-algebra on $\mathcal{S}(X)$, by $\mathcal{A}$. Submechanisms of the system are endowed with a perspective on the event that occurred – on the discrimination performed by the system. Submechanism $\mathfrak{m} \subset \mathfrak{Conn}_X$ in state x_1 specifies the probabilities that various events occurred via its actual repertoire.

Formally, this is expressed as follows. A probability space is a triple $(\mathcal{S}, \mathcal{A}, p)$, where $\mathcal{A}$ is a σ-algebra on $\mathcal{S}$ and p

is a probability measure. A random variable R is then a map to a measure space $\mathcal{T}$ with σ-algebra $\mathcal{B}$:

$$R : \mathcal{S} \to \mathcal{T} \quad \text{such that } R^{-1}(B) \in \mathcal{A} \text{ for all } B \in \mathcal{B}.$$

Events correspond to elements of $\mathcal{B}$, i.e. measurable subsets of $\mathcal{T}$. For example, the probability of the event $B \in \mathcal{B}$ is $p\big(R^{-1}(B)\big)$.

In our case, $\mathcal{S}$ is the state space $\mathcal{S}(X) = \prod_j \mathcal{O}_j$ equipped with the σ-algebra $\mathcal{A}$ introduced above. The set of all probability distributions (repertoires) that can be defined on $\mathcal{S}(X)$ is

$$Q_r(X) = \left\{ p : \mathcal{S}(X) \to \mathbb{R} \,\middle|\, \sum_{x \in \mathcal{S}(X)} p(x) = 1 \text{ and } p \geq 0 \right\} \subset Q(X).$$

This is the subset of qualia space cut out by the two conditions satisfied by probability distributions: they *(i)* are nonnegative and *(ii)* sum to 1. Strictly speaking, we should work with $Q_r(X)$ rather than $Q(X)$ in the paper, however we gloss over this technical distinction since the 'extra points' play no role. Thus, (a subset of) qualia space parametrizes probability distributions on state space.

Every repertoire $p \in Q_r(X)$ induces a unique random variable on the state space by providing it with a unique probability measure:

$$ID : \mathcal{S}(X) \to \mathcal{S}(X) \text{ taking probability space } \big(\mathcal{S}(X), \mathcal{A}, p\big) \text{ to measure space } \big(\mathcal{S}(X), \mathcal{A}\big), \tag{S5}$$

where ID is the identity map. Although the map is constant (and trivial), it defines a different random variable for each repertoire since the probability measure p on $\mathcal{S}(X)$ varies. In other words, the situation is fixed but the perspective – the repertoire specified – varies. Thus, each actual repertoire defines a random variable on the state space by Eq (S5), and for this reason we treat actual repertoires and the random variables they induce as interchangeable.

Returning to the quale, a submechanism $\mathfrak{m}$ in state x_1 specifies actual repertoire $X_0(\mathfrak{m}, x_1)$, which describes the potential states that cause (lead to) x_1 and those that do not. It thus provides a perspective on the event that has occurred: the discriminations performed by the system. Different submechanisms provide different perspectives on the same event, hence the same map ID recurs in all the random variables with different probabilities assigned to states. Typically, the 'larger' the submechanism, the more complete the perspective. The quale geometrically represents all perspectives in a single object.

For example, take the event "the system specified that subset $A \subset \mathcal{S}(X)$ caused (led to) current state x_1". This is an event $A \in \mathcal{A}$. The probability of the event from the perspective of submechanism $\mathfrak{m} \subset \mathfrak{Conn}_X$ is

$$p_{X_0(\mathfrak{m}, x_1)}\Big(ID^{-1}(A)\Big).$$

SI-2 Informational relationships

The camera thought experiment shows that *how* a system discriminates between perturbations is important. Take as wholes, both the camera and a person can discriminate between an enormous variety of different images. However, the camera does not form a single entity; instead there are one million disjoint submechanisms performing simultaneous independent discriminations. The internal structure of the systems (the camera and the human brain) makes a difference. The quale unfolds the differences that make a difference within a system of interlocking submechanisms.

SI-2.1 Levels of analysis

We relate our approach to Marr's three levels of analysis: computational, algorithmic, and implementational [12]. The paper starts at the implementational level – which we would prefer to call mechanistic. In principle, elements could be any idealized (i.e. discrete) piece of the world. Identifying the natural level of mechanistic description (quarks, atoms, neurons or minicolumns?) will be the subject of future work. The paper then develops a language for translating the mechanistic level into a "phenomenological level" that geometrically represents what the system experiences. The phenomenological level can, at a push, be loosely identified with Marr's algorithmic level, with the

proviso that the algorithmic level takes the extrinsic perspective, “How should data be organized and manipulated to carry out the task?”, whereas the phenomenological level takes an intrinsic perspective, “How do I generate information whilst performing the task?”. The algorithmic level is adopted by someone trying to understand how the brain is designed from the outside, whereas the phenomenological level applies to the user on the inside.

The paper takes a positive approach, in contrast to the normative approach more common in cognitive science. I.e. the quale formalism takes the mechanism as given, rather than trying to figure out what it should or could be. The quale formalism is an analytical tool that can be applied *after* a candidate mechanism has been found, not before.

SI-2.2 Posets and lattices keep track of inclusions of parts into wholes

First, we introduce two mathematical tools for keeping track of submechanisms of a system: partially ordered sets and lattices. For details see [13,14]. Partially ordered sets capture the intuitive notion of ordering. This is relevant to the IIT because parts can be ordered according to whether or not they are *included* in one another. Lattices are special partially ordered sets, where any two members have a unique maximum and minimum.

A **poset** (partially ordered set) is a set L equipped with a binary relation $\leq$ satisfying three conditions

1. reflexivity: $x \leq x$
2. antisymmetry: if $x \leq y$ and $y \leq x$ then $x = y$
3. transitivity: if $x \leq y$ and $y \leq z$ then $x \leq z$.

We mention three examples. The real numbers $\mathbb{R}$ form a poset when equipped with $\leq$. Given an arbitrary set Z, we can construct two related posets. The powerset $\mathbf{P}(Z)$, the set of subsets of Z, forms a poset when equipped with inclusion of sets $\subset$. Finally, the set of partitions of Z forms a poset when equipped with refinement (inclusion of partitions, see SI-6 of [5]).

A **lattice** is a poset such that every pair $\{x, y\}$ has a least upper bound (or join) $x \vee y$ and a greatest lower bound (or meet) $x \wedge y$. Each of the three examples above forms a lattice; in the case of the powerset $\vee$ and $\wedge$ correspond to union $\cup$ and intersection $\cap$ respectively.

The set of partitions of X forms a subset of the powerset $\mathcal{L}(X) := \mathbf{P}(\mathfrak{Conn}_X)$. Restricting the operator $\subset$ to the set of partitions induces the refinement relation, so the set of partitions inherits its poset structure from the powerset, and indeed forms a lattice. However, it is not a sublattice since the operations $\cup$ and $\cap$ on the powerset *do not* restrict to give the lattice operations on the set of partitions. For example, it is not necessarily the case that the union $\mathfrak{p}_1 \cup \mathfrak{p}_2$ of two partitions $\mathfrak{p}_1$ and $\mathfrak{p}_2$ is a partition. The $\vee$ operator that induces a lattice structure on the set of partitions does not coincide with $\cup$, so the set of partitions is not a sublattice, although it is a sub-poset, that has a lattice structure. This technical point plays no role in our framework.

The powerset lattice is a **Boolean algebra**. The entire set Z is the greatest element in $\mathbf{P}(Z)$, denoted by $\top$, and similarly the emptyset, denoted by $\bot$, is the smallest element. The notation $\mathbf{0}$ and $\mathbf{1}$ is commonly used for $\bot$ and $\top$, however in the text it would be confusing. Each element x of $\mathbf{P}(Z)$ has a unique complement $\neg x$ satisfying $x \wedge \neg x = \bot$ and $x \vee \neg x = \top$.

A **lattice isomorphism** $f : (L_1, \vee_1, \wedge_1) \to (L_2, \vee_2, \wedge_2)$ is a bijection such that $f(x \wedge_1 y) = f(x) \wedge_2 f(y)$, and similarly for $\vee$. It follows easily that an isomorphism has a unique inverse, also respecting the lattice structures.

SI-2.3 Informational relationships unfold the structure of the discriminations performed by a mechanism

A discrete system is composed of elements and connections. The mechanism of the system depends on the rules implemented by the elements (which may be Boolean or probabilistic functions) and their functional dependencies (how the elements are connected). The set of all possible connections is

$$\mathfrak{Conn}_X = \{c^{ij} | n^i, n^j \in X\}.$$

There is no difference in principle between a system and a subsystem. We keep track of subsystems by their functional dependencies. Thus, a subset $\mathfrak{m}$ of $\mathfrak{Conn}_X$ is referred to as a submechanism of the system. As described

above, every submechanism $\mathfrak{m}$ specifies an actual repertoire, which is computed after imposing maximum entropy noise on all other connections $\neg\mathfrak{m}$ in the system to average out functional dependencies that cannot be accounted for within the submechanism.

To simplify exposition in the main text we abuse notation and use $\mathfrak{Conn}_X$ to refer to connections that actually exist in the system, rather than all possible connections. If c^{ij} is a possible connection in a system that does not physically exist, then the submechanisms $\mathfrak{m}$ and $\mathfrak{m}\cup\{c^{ij}\}$ specify the same actual repertoire for any $\mathfrak{m}$ (connections that do not exist do not contribute to the shape of the quale).

The IIT is concerned with *how* information is generated by interactions in a system. Taking the intrinsic perspective, we relate the discriminations performed by the whole to the discriminations performed by its parts. Thus, we are interested in informational relationships $X_0(\mathfrak{m}, x_1) \to X_0(\mathfrak{m}\cup\mathfrak{r}, x_1)$ between repertoires specified by inclusions $\mathfrak{m} \subset \mathfrak{m}\cup\mathfrak{r}$ of submechanisms. The informational relationship captures how the actual repertoire specified by $\mathfrak{m}\cup\mathfrak{r}$ differs from that specified by $\mathfrak{m}$, and in so doing unfolds an aspect of the quality of the information generated by $\mathfrak{r}$.

Note that it is not meaningful, in terms of the theory, to compare two repertoires $X_0(\mathfrak{a}, x_1)$ and $X_0(\mathfrak{b}, x_1)$ where $\mathfrak{a}$ and $\mathfrak{b}$ are incommensurable: $\mathfrak{a} \not\subset \mathfrak{b}$ and $\mathfrak{b} \not\subset \mathfrak{a}$. To understand how the discriminations performed by $\mathfrak{a}$ and $\mathfrak{b}$ relate it is necessary to take the perspective provided by a larger whole containing both parts such as $\mathfrak{a}\cup\mathfrak{b}$. For example, entanglement of a q-arrow is computed by comparing the repertoire specified by the q-arrow to those specified by its sub-q-arrows, rather than directly comparing the repertoires specified by the sub-q-arrows to one another.

SI-2.4 The quale captures the quality of the information generated by a complex

The quale is the mapping of the lattice of submechanisms into qualia space determined by the actual repertoires specified by a complex in a particular state. It completely characterizes the quality of the information generated by the complex: how the discriminations performed by its mechanism are structured. The above discussion, motivating the definition of the quale, can be summarized as follows:

1. Systems make discriminations (generate information about their prior state) by responding differently to different perturbations, and therefore implicitly categorize the perturbations.
2. The discriminations performed by the (sub)mechanism of a sub(system) is captured by its actual repertoire. The actual repertoire is the simplest invariant that can be associated with a mechanism in a state: it specifies which prior states cause (lead to) the current state, and which do not.
3. The quality of the information generated by a system depends on *how* the information is generated, which depends on how the discriminations performed by the whole relate to the discriminations made by its parts.
4. Extending this argument to its logical conclusion, the points of the quale are given by the actual repertoires specified by every submechanism within a system. The quale therefore geometrically represents the discriminations performed by every submechanism.
5. The actual repertoires are not the primary objects of interest in the quale. Instead, it is the informational relationships between repertoires that capture the quality of the information generated: how the discriminations performed by (sub)submechanisms are integrated into discriminations performed by (sub)mechanisms.
6. Thus, the quale is given by a mapping of the lattice of submechanisms into the qualia space of repertoires. The embedding is simply given interpreting repertoires as points in a high-dimensional space: a probability distribution is nothing more than a collection of numbers. The lattice structure keeps track of inclusion relations.

SI-2.5 Comparison of the quale with graphical models

Graphical models, such as Bayesian networks and factor graphs, provide a formalism for representing dependencies between random variables [15]. For example, a probability distribution may factorize into a product of conditional distributions

$$p(x_{\mathcal{V}}) = \prod_{v\in\mathcal{V}} p\big(x_v|x_{\pi_v}\big),$$

where x_{π_v} is the set of random variables on which x_v is dependent. Uncovering such factorizations is computationally helpful. The factorizations can be represented as a graph where nodes correspond to random variables. This

section explains how and why the quale differs from a graphical model.

A graphical model takes as *given* a set of random variables related to each other by conditional probabilities. Vertices of the graph correspond to events or categories – for example RAIN, DANGER and FOOD – which are modeled as random variables. Edges of the graph correspond to conditional dependencies between random variables; for example $p(\text{DANGER}\,|\,\text{RAIN})$. The graphical model is designed to formalize what is known about the structure of a situation or data-set (e.g. that RAIN increases the probability of DANGER), so that this structural knowledge can be incorporated into an inference algorithm. Typically there is a mechanism somewhere in the background that is responsible for determining the structure of the situation (RAIN makes the road wet, which changes the behavior of a braking car) or producing the data-set. However, the conditional probabilities are the primary objects of study, and the mechanism is forgotten once the conditional probabilities have been somehow extracted.

The quale takes as *given* a mechanism that is in a state. Vertices of the quale correspond to submechanisms, each of which specifies an actual repertoire by discriminating between potential inputs. Edges of the quale correspond to inclusions of submechanisms, which are geometrically realized as q-arrows (informational relationships). Thus, each vertex represents a subperspective on the information generated by the whole, and informational relationship capture the difference between perspective and subperspectives. The quale is designed to unfold how the quality of the information generated by the system reflects the compositional structure of the mechanism.

The mechanism implicitly divides the world into categories by responding differently to different inputs. Entanglement and concepts make these implicit categories explicit, by identifying indecomposable informational relationships within the quale. It has no analogue in graphical models, since in this case the concepts are built into the model. Graphical models beg the question that the quale seeks to answer: Where do the concepts come from?

SI-2.6 The information generated about elements across connections can be quantified

The quale geometrically represents how a system generates information. We are interested in quantifying phenomenologically meaningful properties of the quale. To this end, we observe that elements and connections play distinct roles in the framework. Elements produce outputs, which the mechanism of the system discriminates between. Connections represent functional dependencies within the system and combinations of connections label submechanisms of the system. Thus, information is generated about elements across connections. A fruitful approach to quantifying aspects of the quale is to analyze the interplay between elements and connections.

The lattice of submechanisms $\mathcal{L}(X)$ keeps track of all possible causal interactions within the system X, and we use it to specify the quale. Subsets of the set of elements form a second Boolean lattice $E(X)$. Each member M of $E(X)$ defines a projection operator π_M that maps repertoires in qualia space onto a linear subspace by marginalizing. $\mathcal{L}(X)$ and $E(X)$ are used to track submechanisms and subrepertoires of X respectively.

Suppose we have sets of connections $\mathfrak{m}$ and $\mathfrak{r}$, and a partition $\mathcal{P} = \{M^k\}$ of elements of X; and a collection $\mathcal{R} = \{\mathfrak{r}^k\}$ of subsets of $\mathfrak{r}$. Define generalized effective information as

$$ei\Big(X_0(\mathfrak{m}, x_1) \rightarrow X_0(\mathfrak{m}\cup\mathfrak{r}, x_1)/\mathcal{P},\mathcal{R}\Big) = H\left[X_0(\mathfrak{m}\cup\mathfrak{r}, x_1)\,\middle\|\,\prod_{M^k\in\mathcal{P},\mathfrak{r}^k\in\mathcal{R}} M_0^k(\mathfrak{m}\cup\mathfrak{r}^k, x_1)\right],$$

where $M_0^k(\mathfrak{m}\cup\mathfrak{r}^k, x_1) = \pi_{M^k}\big[X_0(\mathfrak{m}\cup\mathfrak{r}^k, x_1)\big]$. Integrated information and entanglement are two ways of analyzing how the connection $\mathcal{L}(X)$ and projection $E(X)$ lattices are coupled by interactions in the system.

	integrated information (ϕ)	entanglement (γ)
$\mathfrak{m}$	$\perp$	arbitrary
$\mathfrak{r}$	$\top$	arbitrary
$\mathfrak{r}^k$	connections with *source* and *target* in M^k	connections with *source* in M^k

Integrated information quantifies how the cost (in bits) of decomposing a system is into subsystems. Entanglement quantifies the cost (in bits) of decomposing a q-arrow into sub-q-arrows.

Both measures are answers to special cases of the question: How do the submechanisms of X structure its subrepertoires? Which connections make a difference to the information generated about which elements?

SI-3 Integrated information

The camera thought experiment implies that the information generated by a system is experienced to the extent that it is generated by a single entity, which is quantified as integrated information (ϕ). In the case of the digital camera a large amount of information is produced but $\phi = 0$ since there is no entity – no point of view – that integrates the information into a whole. ϕ quantifies the cost in bits of decomposing the information generated by a system into that generated independently by its parts; the extent to which "the whole is more than the sum (or rather the product) of its parts". Integrated information is discussed in detail for discrete systems in [5]. This section explains how integrated information relates to the geometry of the quale.

SI-3.1 The MIP is the partition of the system that does the least damage to the information generated

ϕ measures the information generated by a q-arrow in the quale: the informational relationship $X_0(\mathfrak{p}^{MIP}, x_1) \to X_0(\top, x_1)$ between the actual repertoire specified by connections within the MIP and the actual repertoire specified by the entire system. The MIP is found by applying a version of Ockham's razor, "entities must not be multiplied beyond necessity". The cost of "multiplying parts" within a system is quantified as the difference the parts make to the discriminations the system performs: the entropy of the actual repertoire of the whole relative to the parts taken independently. The MIP is the partition of the system that has the lowest cost (after normalizing). It is the partition that does the least damage to the information generated. For example, Ockham's razor effortlessly slices the digital camera into a million separate entities without altering the information generated at all. ϕ thus quantifies the cost in bits to decomposing the perspective provided by the whole into a collection of independent subperspectives.

Every partition provides a subperspective on the discriminations performed by the whole; how the discriminations are seen from the collection of the parts in the partition. Effective information $ei\big(X_0(\mathfrak{p}, x_1) \to X_0(\top, x_1)\big)$ quantifies how the whole differs from those parts. Every q-arrow is a strut, providing the quale with "breathing room" relative to a given decomposition. The ϕ q-arrow is then privileged, since it captures how far the system is from decomposing into the MIP; the parts that are closest to capturing the information generated by the whole.

SI-3.2 Comparison of integrated information with multivariate mutual information

Multivariate mutual information is defined as

$$I(X_1; \cdots ; X_n) = \sum_{k \leq n} (-1)^{k+1} \sum_{X \subset (X_1, \ldots, X_n) \text{ s.t. } |X|=k} H(X).$$

Unfortunately, as observed by Amari [16], multivariate mutual information can be negative, which is problematic for two reasons. First, it is unclear how to interpret negative information. Second, the 'canceling out' of terms makes the interpretation of nonnegative values unclear as well. For example, $I = 0$ implies that the sum of even ($2k$) terms has equal magnitude to the sum of odd ($2k + 1$) terms. This is not a useful criterion. By contrast, effective information between two repertoires is always nonnegative, and is zero if and only if the repertoires are identical. Thus, effective information can be meaningfully interpreted as a measure of the difference between two repertoires.

It could be argued that effective information is conceptually simpler than multivariate mutual information. First, notice that multivariate mutual information is expressed as an alternating sum of entropies and that each entropy can be expressed as an *average* of relative entropies, which we take to be the more fundamental concept. Neither the alternating sum nor taking an average of entropies makes sense when taking the perspective of a system in a state. Second, there is a trade-off between conceptual and practical simplicity. Probability distributions on neuronal activity are simple to obtain in practical terms, but difficult to make sense of conceptually since the relationship between the measuring apparatus and the actual neuronal activity is difficult to pin down precisely. By contrast, the actual repertoire is conceptually extremely simple (the alternatives ruled out by the mechanism), but difficult to compute in practice since: *(i)* neuronal mechanisms are not well understood and *(ii)* there is a combinatorial explosion in the number of alternatives as a function of the size of the mechanism.

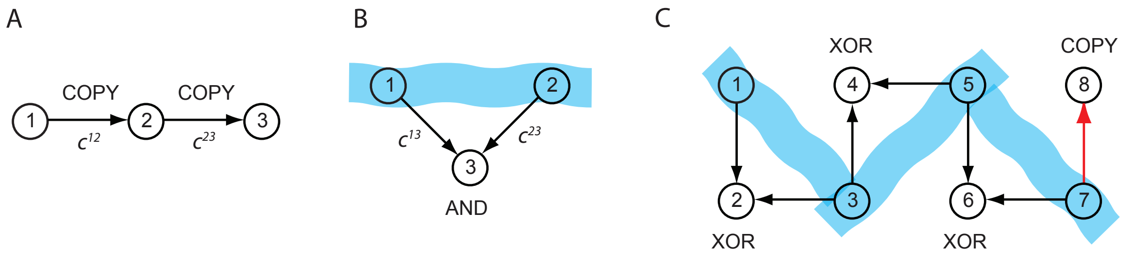

Figure S2: Entanglement in deterministic systems. (A): a disentangled system, the prior state of n^1 is specified independently of n^2. (BC): tangled systems. Bits in the actual repertoire that are tangled are linked by cyan lines. The red connection c^{78} is discussed in the text.

SI-3.3 Comparison of integrated information with geometric decompositions of probability distributions

It is instructive to compare ϕ and the MIP with an alternative geometric approach to decomposing a probability distribution.

Motivated (presumably) by a desire to understand correlations in neuronal activity, Amari found a family of measures quantifying how far a probability distribution is from decomposing into a product [16]. Specifically, he measures the divergence between the distribution and its projection onto a subspace of product distributions, where the product is taken over some partition. In [17], Nakahara and Amari apply the measure to decompose spiking activity into pairwise, triple-wise and higher order interactions. This is a powerful method for analyzing how information about the environment is encoded in neural activity. This formalism has been elegantly extended in [18] to provide a family of geometrically motivated complexity measures.

There are three main differences we wish to emphasize between our approach and Amari's. First, we take the intrinsic perspective since we are interested in understanding the experience generated by the system. [17] takes the perspective of an external observer who watches and records the activity of a population of neurons. Neurons do not take this detached perspective: they cannot record activity and then analyze it later. Instead they respond differently to different activity patterns, implicitly classifying them. Second, Amari is concerned with understanding correlations in neuronal activity, whereas we are concerned with the *causal* interactions that result in a particular output. To capture the mechanisms perspective on causal interactions in the system we have introduced the notion of an actual repertoire, which has no analogue in [16, 17].

Third, and relatedly, our approach to decomposing a repertoire into parts (the quale) differs from that of Amari due to the differing perspective (extrinsic versus intrinsic) taken. Amari takes a distribution and projects it onto a product subspace. Notice however that it is *Amari* that does the projecting, since he (and others) find it useful to decompose distributions into independent components: the orthogonal projections provide a convenient and useful way for an external observer to divide up the neuronal activity. In the paper, we instead take each submechanism of the system and ask how *the submechanism* discriminates between potential prior states. We therefore project onto submechanisms as opposed to subspaces. Nevertheless, the actual repertoire specified by the MIP is a product distribution; however, it will not in general coincide with the orthogonal projection constructed of [16], since they seek to answer different questions. Amari's projection provides the product distribution closest to the entire distribution. The actual repertoire specified by the MIP provides the product distribution specified by the parts themselves. In this way, we uncover the structure of the information from the system's perspective.

SI-4 Entanglement

We briefly relate entanglement to more familiar notions: deviation from orthogonality (or independence), integrated information, and the (different) notion of entanglement in quantum mechanics. We also show that nonzero entanglement is necessary for a collection of components to generate more information than their sum, taken individually; and that strictly feedforward networks are always disentangled.

SI-4.1 Motivation and relation to previous work

Figure S2A depicts a chain of three elements, each copying its prior input. Effective information generated by the system as a whole is $ei(A_0 \xrightarrow{\mathfrak{Conn}_A} a_1) = 2$ bits. Notice that the two bits are independent of one another, the first is specified by the causal interaction across connection c^{12}, and the second across c^{23}. The system as a whole is integrated, in that $\phi(a_1) = 1$ bit, but its two connections generate two distinct bits of information that, in a memoryless system, have nothing to do with each other: the q-arrow corresponding to the union of the two connections decomposes into two independent (orthogonal) q-arrows corresponding to each connection. Entanglement is $\gamma\big(X_0(\bot, x_1) \to X_0(\top, x_1)\big) = 0$ bits, with $\mathcal{P}^{min} = \{1|23\}$. Thus, the interactions in the system decompose into two independent ("orthogonal") sub-q-arrows, which are context independent.

Consider now the system in panel B, which also contains three elements, but includes an *AND*-gate. Effective information for the q-arrow corresponding to the union of the two connections is .4 bits. However, in this case the effective information is generated by causal interactions across both c^{13} and c^{23} simultaneously and cannot be decomposed into independent (orthogonal) q-arrows. Specifically, the actual repertoire on elements n^1 and n^2 is $p(00, 01, 10, 11) = (\frac{1}{3}, \frac{1}{3}, \frac{1}{3}, 0)$, which is not a product distribution, so *it is not possible to specify the possible prior state of either element independently of the other.* The actual repertoire on the two elements is tangled. Entanglement arises from taking an intrinsic perspective on physical interactions. The system does not have perfect information regarding its prior state, and the information that it does have is generated across an enmeshed set of connections, resulting in a tangled actual repertoire: $\gamma\big(X_0(\bot, x_1) \to X_0(\top, x_1)\big) = 1$ bit, with a minimum partition again given by $\mathcal{P}^{min} = \{1|23\}$. Breaking the system into sub-q-arrows carries a 1 bit cost. The sub-q-arrows do not generate information independently, and are not truly distinct.

Fig. S2C depicts a system of *XOR*-elements, where each element is silent at time 1. Let $\mathfrak{m}$ contain all connections in the system except c^{78}. The actual repertoire $X_0(\mathfrak{m}, x_1)$ specifies that the diagonal was either all on or all off at time 0. Entanglement is $\gamma\big(X_0(\bot, x_1) \to X_0(\mathfrak{m}, x_1)\big) = 1$ bit. Now suppose we engage connection c^{78}. The actual repertoire $X_0(\top, x_1)$ specifies that elements $n^1, \ldots, n^7$ were silent at time 0. The causal interaction across c^{78} *collapses* the uncertainty in repertoire $X_0(\mathfrak{m}, x_1)$, resulting in a distribution specifying a single perturbation with $p = 1$. After engaging connection c^{78} it is no longer obvious from the actual repertoire $X_0(\top, x_1)$ that the bits in the system are tangled, since all the bits are specified.

To deal with situations like that in Fig. S2C, entanglement should depend on *how* the actual repertoire of the system is generated, rather than simply depending on the final product. It is not enough to consider the actual repertoire generated by the whole system; it is necessary to explicitly analyze how the submechanisms of the system specify the repertoire. Entanglement $\gamma\big(X_0(maxH) \to X_0(\top, x_1)\big) = 1$ bit, since the cyan interactions do not decompose into independent components.

To illustrate the point, we compare entanglement with a possible alternative definition that produces an unsatisfactory result when applied to Fig. S2C. As shown in Eq (S1) of [5], effective information can be rewritten as a sum of simple subexpressions:

$$\phi(x_1) = ei(S_0 \to s_1/\mathcal{P}^{min}) = \sum_k H\left[M_0^k(\top, s_1) \,\big\|\, M_0^k(\mathfrak{p}^k, \mu_1^k)\right] + H\left[S_0(\top, s_1) \,\Bigg\|\, \prod_{M^k \in \mathcal{P}^{min}} M_0^k(\top, s_1)\right].$$

The $H\left[M_0^k(\top, s_1) \,\big\|\, M_0^k(\mathfrak{p}^k, \mu_1^k)\right]$ terms quantify the information generated by the whole about the prior state of M^k over and above the information generated internally by the part. The second term is the integration, in the sense of [19], of the repertoire of the whole. Integration, not to be confused with integrated information, generalizes mutual information to partitions of more than two parts. It quantifies the extent, in bits, to which the actual repertoire of the whole cannot be described as the product of its projections (marginalizations) onto the parts.

However

$$H\left[S_0(\top, s_1) \,\Big\|\, \prod M_0^k(\top, s_1)\right] \tag{S6}$$

is not suitable as a measure of entanglement, since it is blind to how the bits in Fig. S2C are jointly specified by the *XOR*-gates in the system: Eq (S6) yields zero when applied to Fig. S2C. Entanglement is therefore defined by modifying the expression to expose *how* $X_0(\top, x_1)$ is specified by uncovering the interactions between

submechanisms of the system. Entanglement of a quale $Q(s_1)$ is

$$\gamma\Big(S_0(maxH) \to S_0(\top, s_1)\Big) = H\left[S_0(\top, s_1) \,\Bigg\|\, \prod_{M^k \in \mathcal{P}^{min}} M_0^k(\mathfrak{r}^k, s_1)\right], \tag{S7}$$

where $\mathfrak{r}^k$ contains the connections in S sourcing from M^k. Eq (S7) quantifies the extent to which the actual repertoire of the whole does not reduce to the product of the actual repertoires specified by the submechanisms of the parts. By contrast, Eq (S6) quantifies the extent to which the actual repertoire does not reduce to a product of subrepertoires (obtained by projecting onto subsystems), without regard for the differential contribution of the submechanisms.

SI-4.2 Interpreting entanglement geometrically

The hypotenuse of a right-angled triangle has length $\sqrt{a^2+b^2}$, where a and b are the lengths of the other two sides, assuming the triangle is drawn on a flat Euclidean plane. Pythagoras' formula can be generalized to define the distance between two points x and y in Euclidean n-space as $d(x,y) = \sqrt{\sum_i (x_i - y_i)^2}$. In non-Euclidean geometry, the length of the shortest geodesic connecting two points will not in general be described by Pythagoras' formula.

Suppose we live in a discrete world, consisting of a finite set of points equipped with a metric. A natural question to ask is: What is the geometry of the world? If the world consists of vertices of an n-cube, then for any three consecutive vertices x, y, z:

$$d(x,z) = \sqrt{d(x,y)^2 + d(y,z)^2} \tag{S8}$$

Similar equations relate non-consecutive vertices. If there are points in the world that are not related by (S8) then the world is not an n-cube. Deviations from Pythagoras' formula reflect deviations from orthogonality in the geometry of the world. The difference

$$diff = d(x,z) - \sqrt{d(x,y)^2 + d(y,z)^2} \tag{S9}$$

measures the local deviation from orthogonality.

Entanglement can be thought of as a non-metric[1] analog of $diff$ as follows. A system in which every connection generates information regardless of every other connection generates the analog of the n-cube. In such a system, given any triple $p \subset q \subset r$ we have

$$H[r\|p] = H[r\|q] + H[q\|p], \tag{S10}$$

providing the non-metric analog of Pythagoras [20]. The geometric notion of orthogonality corresponds to independence in information theory. Entanglement measures the deviation from this ideal situation. One way to generalize (S9) is by subtracting the RHS (right hand side) of (S10) from the LHS, computing a difference of entropies. We prefer to directly compare repertoires (see SI-8d of [5]) and use the fact that, in our setup, $r = X_0(\mathfrak{m} \cup \mathfrak{r}, x_1)$ is specified by a mechanism $\mathfrak{m} \cup \mathfrak{r}$ that subsumes the mechanism $\mathfrak{m}$ specifying $p = X_0(\mathfrak{m}, x_1)$.

If the connections in $\mathfrak{r}$ generated information independently of one another, over and above those in $\mathfrak{m}$, there would be a partition of $\mathcal{P} = A^1|A^2$ of X such that

$$X_0(\mathfrak{m} \cup \mathfrak{r}, x_1) = A_0^1(\mathfrak{m} \cup \mathfrak{r}^1, x_1) \cdot A_0^2(\mathfrak{m} \cup \mathfrak{r}^2, x_1)$$

where $\mathfrak{r}^1$ is the connections in $\mathfrak{r}$ that have their source in A^0, and similarly for $\mathfrak{r}^2$. Entanglement measures the difference (relative entropy) between the repertoire as it is specified, and the repertoire as it would be specified in the ideal "independent" system

$$\gamma\Big(X_0(\mathfrak{m}, x_1) \to X_0(\mathfrak{m} \cup \mathfrak{r}, x_1)/\mathcal{P}\Big) = H\Big[X_0(\mathfrak{m} \cup \mathfrak{r}, x_1) \,\Big\|\, A_0^1(\mathfrak{m} \cup \mathfrak{r}^1, x_1) \cdot A_0^2(\mathfrak{m} \cup \mathfrak{r}^2, x_1)\Big]$$

and is thus the information-theoretic analog of (S9).

It is helpful to picture unentangled q-arrows as a parallelogram, since the divergences of opposing sides are equal. Entanglement then measures the deviation from a parallelogram-like structure: how far the geometry is from Pythagorean. Strictly speaking this is not accurate, since divergences are not lengths: the geometry is non-metric and so the comparison to a parallelogram is only an analogy.

[1] Qualia space can be equipped with a non-Euclidean Fisher metric, an avenue we do not pursue here; see [20].

SI-4.3 Quantum entanglement

Quantum entanglement (henceforth QE) occurs when the states of two objects are linked so that the state of neither object can be described independently of the other. Entanglement in the sense introduced here formally resembles QE (hence the name), but it is *not* a quantum phenomenon. In this section we briefly flesh out the formal similarity between the two measures.

First, recall how qualia space was defined. We began with a discrete system of n (binary) elements. State space is then the n-dimensional space (an axis per element) of possible states of the system. Qualia space was then defined as the set of possible real-valued functions on state space. A repertoire or probability distribution is a real-valued function with all values non-negative that sums to 1.

The analog of the qualia space in quantum mechanics is the Hilbert space of quantum states: complex-valued (wave) functions defined on the possible states of the system. There are many measures of QE. We describe the simplest: von Neumann mutual information. Given a quantum state (vector in Hilbert space) ρ_X of a combined system $X = AB$, the von Neumann mutual information is [21]

$$I_N(\rho_A : \rho_B; \rho_X) = Tr(\rho_X \ln \rho_X) - Tr(\rho_A \ln \rho_A) - Tr(\rho_B \ln \rho_B). \tag{S11}$$

Here Tr is a sum over coordinates. Von Neumann mutual information is thus a straightforward generalization of mutual information.

Replacing vectors in Hilbert space with repertoires in qualia space, Eq (S11) can be rewritten as a relative entropy:

$$H\Big[X_0(\top, x_1)\, \Big\|\, A_0(\top, x_1) \cdot B_0(\top, x_1)\Big] \,,$$

which computes mutual information, and is identical to Eq (S6). See SI-4.1 for a discussion of how Eq (S6) relates to entanglement.

SI-4.4 A disentangled system generates effective information equal to the sum of its minimal q-arrows

Suppose $\gamma\big(X_0(maxH) \to X_0(\top, x_1)\big) = 0$, where the minimum information partition is a bipartition (the argument extends easily to general partitions). It follows that

$$X_0(\top, x_1) = M_0^0(\mathfrak{r}^0, x_1) \cdot M_0^1(\mathfrak{r}^1, x_1).$$

Expanding the formula for effective information we obtain

$$\begin{aligned} ei\Big(X_0(\top, x_1)\Big) &= H\left[X_0(\top, x_1) \,\|\, X_0(maxH)\right] \\ &= H\left[M_0^0(\mathfrak{r}^0, x_1) \cdot M_0^1(\mathfrak{r}^1, x_1) \,\big\|\, X_0(maxH)\right] \\ &= H\left[M_0^0(\mathfrak{r}^0, x_1) \,\big\|\, M_0^0(maxH)\right] + H\left[M_0^1(\mathfrak{r}^1, x_1) \,\big\|\, M_0^1(maxH)\right] \\ &= ei\Big(X_0(\mathfrak{r}^0, x_1)\Big) + ei\Big(X_0(\mathfrak{r}^1, x_1)\Big). \end{aligned}$$

Thus it is necessary, but not sufficient, that $\gamma > 0$ for the whole to generate more effective information than the sum of the minimal interactions.

SI-4.5 Entanglement is zero in strictly feedforward networks

We show that $\gamma\big(X_0(maxH) \to X_0(\top, x_1)\big) = 0$ for any strictly feedforward network.

In any feedforward network there is an element or group of elements, B, at the top of the network, with no back or lateral connections to other elements. These elements generate information about other elements, but have no outgoing connections, so their state cannot be specified by the rest of the system. Introduce bipartition $A|B$, where A contains all other elements in the network. Let $\mathfrak{a}$ be all connections sourcing from $\mathfrak{a}$ and similarly for $\mathfrak{b}$. Since $\mathfrak{b}$ contains no connections, it follows trivially that $\mathfrak{a}$ and $\mathfrak{b}$ are not tangled, as desired. For this purpose we consider $\mathfrak{Conn}_X$ as the set of all possible connections in X the non-existent back-connections are not entangled.

Thus, in any strictly feedforward network, there are bits of information about the prior state that are not entangled.

For example, in the *AND*-gate discussed in Fig. 4A of the main text, the prior states of elements n^1 and n^2 are entangled, but the prior state of element n^3 is completely unspecified and therefore not entangled with the other two. This feature (the bits at the top of the network remaining disentangled) will occur in any strictly feedforward network.

SI-4.6 Entanglement > 0 implies integrated information > 0

We show that

$$H\Big[X_0(\top, x_1)\Big\|A_0(\mathfrak{p}^1, a_1)\cdot B_0(\mathfrak{p}^2, b_1)\Big] = 0 \implies H\Big[X_0(\top, x_1)\Big\|A_0(\mathfrak{a}, x_1)\cdot B_0(\mathfrak{b}, x_1)\Big] = 0,$$

where $\mathfrak{a}$ and $\mathfrak{b}$ contain the connections with source in parts A and B respectively; and $\mathfrak{p}^1$ and $\mathfrak{p}^2$ contain the connections with source and target in A and B respectively.

Since $H[p\|q] = 0$ if and only if $p = q$, the problem reduces to showing

$$H\Big[A_0(\mathfrak{p}^1, a_1)\cdot B_0(\mathfrak{p}^2, b_1)\Big\|A_0(\mathfrak{a}, x_1)\cdot B_0(\mathfrak{b}, x_1)\Big] = 0\ ,$$

Let $\mathfrak{a} = \mathfrak{p}^1 \cup \mathfrak{r}$, where $\mathfrak{r}$ is the set of all connections going from A to B, and $\top = (\mathfrak{p}^1 \cup \mathfrak{r}) \cup (\mathfrak{p}^2 \cup \mathfrak{s})$, where $\mathfrak{s}$ is the set of all connections going from B to A. We are now reduced to showing that

$$A_0(\mathfrak{p}^1, x_1) = A_0(\mathfrak{p}^1 \cup \mathfrak{r}, x_1) \text{ given } A_0(\mathfrak{p}^1, x_1) = A_0\Big((\mathfrak{p}^1 \cup \mathfrak{r}) \cup (\mathfrak{p}^2 \cup \mathfrak{s}), x_1\Big), \text{ and similarly for } B_0.$$

We argue as follows. Connections in $\mathfrak{p}^2$ and $\mathfrak{s}$ do not contribute to specifying A, since they have sources in B, and the actual repertoire of the whole is the product of the actual repertoires of the two parts. Thus,

$$A_0\Big((\mathfrak{p}^1 \cup \mathfrak{r}) \cup (\mathfrak{p}^2 \cup \mathfrak{s}), x_1\Big) = A_0(\mathfrak{p}^1 \cup \mathfrak{r}, x_1).$$

Conversely, it follows that if the quale is tangled, $\gamma\Big(X_0(maxH) \to X_0(\top, x_1)\Big) > 0$, then the system generates integrated information, $\phi(x_1) > 0$. Thus, every tangled q-arrow is contained in a complex.

SI-5 Context-dependency in the quale

The quale unfolds all the informational relationships generated by the submechanisms in a complex of elements. We show how the structure of the quale can be used to analyze the context-dependence of the information generated.

SI-5.1 Inserting and removing context

In the main text, we showed how a given submechanism $\mathfrak{r} \subset \mathfrak{Conn}$ defines a q-fold in the quale $Q(x_1)$. The q-fold is the set of all informational relationships generated by the connections in $\mathfrak{r}$ in all contexts:

$$\Big\{X_0(\mathfrak{m}, x_1) \to X_0(\mathfrak{m} \cup \mathfrak{r}, x_1)\ \Big|\, \mathfrak{m} \in \mathcal{L}(X)\Big\}\ .$$

It is useful to have a precise notation for moving between informational relationships in the q-fold. For example, the informational relationships generated by $\mathfrak{r}$ in the null context $X_0(maxH) \to X_0(\mathfrak{r}, x_1)$ and full context $X_0(\neg\mathfrak{r}, x_1) \to X_0(\top, x_1)$ are of particular interest. The structure of the two informational relationships is given by the subqualia $\downarrow X_0(\mathfrak{r}, x_1)$ and $\uparrow X_0(\neg\mathfrak{r}, x_1)$.

The two subqualia, in the null and full contexts are related as follows. Each q-arrow in $X_0(\mathfrak{a}, x_1) \to X_0(\mathfrak{a}\cup\mathfrak{b}, x_1) \in \downarrow X_0(\mathfrak{r}, x_1)$ maps to to a q-arrow in $X_0(\neg\mathfrak{r} \cup \mathfrak{a}, x_1) \to X_0(\neg\mathfrak{r} \cup \mathfrak{a} \cup \mathfrak{b}, x_1) \in \uparrow X_0(\neg\mathfrak{r}, x_1)$, where the latter q-arrow is determined by adding the context $\neg\mathfrak{r}$ to the former q-arrow. Similarly, q-arrows in the full context subquale can be mapped to the null context quale by stripping out the context of $\neg\mathfrak{r}$.

The operations of inserting and removing context are formalized as follows. For each subset $\mathfrak{r} \subset \mathcal{L}(X)$ we have a pair $(g^*_\mathfrak{r}, g^\mathfrak{r}_*)$ of maps from the lattice $\mathcal{L}(X)$ to itself, where

$$g^*_\mathfrak{r} : \mathcal{L}(X) \to \mathcal{L}(X) : \mathfrak{m} \mapsto \mathfrak{m} \cap \mathfrak{r} \quad \text{and} \quad g^\mathfrak{r}_* : \mathcal{L}(X) \to \mathcal{L}(X) : \mathfrak{m} \mapsto \mathfrak{m} \cup \neg\mathfrak{r}. \tag{S12}$$

The map $g_{\mathfrak{r}}^*$ pulls lattice element $\mathfrak{m}$ back into the down-set $\downarrow\neg\mathfrak{r}$. The pullback map $g_{\mathfrak{r}}^*$ acts to strip out the context provided by connections in $\mathfrak{r}$. For example, applying the pullback map to an informational relationship of the form $X_0(\mathfrak{m}, x_1) \to X_0(\mathfrak{m}\cup\mathfrak{a}, x_1)$ produces new informational relationship $X_0(\mathfrak{m}\cap\neg\mathfrak{r}, x_1) \to X_0\big((\mathfrak{m}\cup\mathfrak{a})\cap\neg\mathfrak{r}, x_1\big)$. In this way the two informational relationships (before and after stripping out the context $\mathfrak{r}$) can be directly compared. Similarly, the map $g_*^{\mathfrak{r}}$ pushes element $\mathfrak{m}$ forward onto the up-set $\uparrow\neg\mathfrak{r}$. The pushforward map can be used to analyze the effects of adding context $\neg\mathfrak{r}$ to a measurement. The pair $(g_{\mathfrak{r}}^*, g_*^{\mathfrak{r}})$ of maps is referred to as a Galois connection [22].

SI-5.2 Semantics

Informational relationships and concepts have counterparts in semantics. In the main text, an informational relationship is defined as a q-arrow $X_0(\mathfrak{m}, x_1) \to X_0(\mathfrak{m}\cup\mathfrak{a}, x_1)$ in the quale. The definition can be compared to the notion of secondary intension in two-dimensional semantics [23], where the meaning of a sentence is described in terms of its primary and secondary intensions. The secondary intension of a sentence is given by the set of possible worlds in which it is true. Meaning is determined by considering all counterfactuals. For example, the meaning of "That car is old" is that which is constant in all possible worlds where the sentence is true. In particular, since the sentence remains true if the color of the car is changed, we see that color is irrelevant to its meaning. Formally, the secondary intension of a sentence is a map from counterfactual worlds W_C to truth values $v_C : W_C \to \{0,1\}$. Equivalently, the secondary intension corresponds to pair $\big(W_C, v_C^{-1}(1)\big)$, consisting of the set of possible worlds, and the distinguished subset where the sentence is true.

The IIT grounds semantics in the action of a mechanism. The current state x_1 plays the role of a sentence. The meaning of x_1 is generated by the mechanism, and is given by the relationship between two repertoires, i.e. by a q-arrow. For example, in q-arrow $X_0(maxH) \to X_0(\top, x_1)$ the set of counterfactual words W_C corresponds to the potential repertoire of equally weighted possible states. The subset $v_C^{-1}(1)$ of worlds where the sentence is true corresponds to perturbations with non-zero probability in the actual repertoire. Thus, we replace the difficult to define notion of a "possible world" with the concrete set of possible outputs that can be distinguished by the system. Further, truth values are not assigned abstractly, but replaced with perturbations concretely specified by the processing of the system, which separates those alternatives that cause (lead to) the output, and cannot be distinguished by the mechanism, from those alternatives that do not. Whereas in possible world semantics the meaning of a sentence corresponds to the possible worlds in which it is true; in a discrete system the meaning of an output corresponds to the potential alternatives it specifies.

In the main text, a q-fold is defined as the set of all informational relationships generated by a submechanism $\mathfrak{r}$ in all contexts. The definition can be related to primary intension in two-dimensional semantics. Primary intension captures the context of a sentence by considering actuals rather than counterfactuals: which car is "that car"? The context is the *actual* car referred to. Given a primary intension, the meaning of the sentence is again provided by the secondary intension: "given that *that* is the car in question, under what conditions does it count as old?" The context is introduced prior to considering counterfactuals. Formally, the two dimensional intension of a sentence is the map from actual worlds W_A to secondary intensions:

$$v : W_A \to (W_C \to \{0,1\}). \tag{S13}$$

Each primary intension (actual context) is mapped to a secondary intension, where the counterfactuals determine the meaning given the context. Informally, $W_A \to (\;\bullet\;)$ sets up the context by specifying what a car is, and which car is *that* car; and $W_C \to \{0,1\}$ specifies whether or not *that* car is old.

The lattice $\mathcal{L}(X)$ of submechanisms of $\mathfrak{Conn}_X$ provides a concrete way of keeping track of all possible contexts in a system. Consider a set $\mathfrak{m}$ of connections in $\mathfrak{Conn}_X$. The secondary intension generated by the submechanism $\mathfrak{m}$ in isolation is represented by $X_0(maxH) \to X_0(\mathfrak{m}, x_1)$. Note, however that we can vary the context in which $\mathfrak{m}$ is engaged. Recall the pushforward (S12)

$$g_*^{\neg\mathfrak{r}} : \mathcal{L}(X) \to \mathcal{L}(X) : \mathfrak{m} \mapsto \mathfrak{m}\cup\mathfrak{r}.$$

By considering the action of the pushforward map on all contexts (q-fold) we capture the two dimensional intension of the submechanism $\mathfrak{r}$:

$$\begin{aligned} G_*^{\neg\mathfrak{r}} : \mathcal{L}(X) &\to \textbf{Information-Relations} \\ \mathfrak{m} &\mapsto \Big[X_0(\mathfrak{m}, x_1) \to X_0(\mathfrak{m}\cup\mathfrak{r}, x_1)\Big] \end{aligned} \tag{S14}$$

The image of map $G_*^{\neg\mathfrak{r}}$ is the q-fold generated by submechanism $\mathfrak{r}$: the set of all informational relationships generated by $\mathfrak{r}$ in all possible contexts. The q-fold of $\mathfrak{r}$ should be thought of as the information-theoretic instantiation of (S13).

Notice that $G_*^{\neg\mathfrak{r}}$ does not range over all imaginable contexts, but rather, by ranging over submechanisms of the system, completely characterizes the *actual* context in which the $\mathfrak{r}$-connections engage. The context structures the counterfactuals ruled out by interactions within X.

In the main text, a concept is defined as an informational relationship with $\gamma\Big(X_0(\mathfrak{m}, x_1) \to X_0(\mathfrak{m}\cup\mathfrak{r}, x_1)\Big) > 0$. Thus, a concept is an informational relationship that does not decompose into a collection of independent sub-q-arrows. A similar phenomenon arises in semantics, where phrases do not necessarily decompose into independent components (words). For example, the word "old" has a different meaning – rules out different time frames – in the phrases "old car" and "old planet".

Crucial to the IIT is considering not merely the q-arrow $X_0(\mathfrak{m}, x_1) \to X_0(\mathfrak{n}, x_1)$, but its structure, given by sublattice $X_0(\downarrow\mathfrak{n} \cap \uparrow\mathfrak{m}, x_1)$. By analyzing the structure we are able to investigate how sub-q-arrows within a q-arrow interact, potentially forming indivisible concepts. An interesting measure of the complexity of concepts (in the special case where they are generated by a single Boolean element) is given in [24]. Roughly, the measure quantifies the minimum description length of the concept. By contrast, entanglement measures the interdependence of the sub-q-arrows that generate a concept.

SI-6 Resolutions

The structure of a quale is complicated, even in fairly small systems. Before studying qualia in more interesting examples, it is necessary to develop tools to simplify analysis. As a first step in this direction, we introduce the notion of a resolution.

A **resolution** is a partition of $\mathfrak{Conn}_X$. In the main text, we frequently found it convenient to represent the quale of a system without showing every single q-arrow. Coarser resolutions make analysis and visualization simpler, at the price of ignoring finer structure. To minimize the damage, resolutions should respect the structure (entanglement) of the q-arrows.

Example. Take a system X and consider resolution $\mathcal{R} = \{\mathfrak{v}|\mathfrak{a}\}$ of $\mathfrak{Conn}$, where $\mathfrak{v}$ contains connections in the visual system and $\mathfrak{a}$ connections in the auditory system; the system does nothing but see and hear. Write $Q_{\mathcal{R}}(x_1)$ for the view on the quale at this – very coarse – resolution:

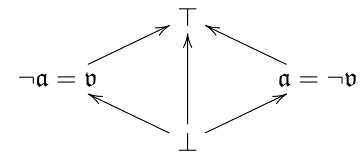

From the coarse-grained quale $Q_{\mathcal{R}}(x_1)$ one can compute:

1. The "raw" contribution of each subsystem to experience: $\neg\mathfrak{v} \to \top$
2. The influence of context on each subsystem: compare $\bot \to \mathfrak{v}$ to $\neg\mathfrak{v} \to \top$
3. Entanglement between the subsystems: how far is $\top$ from product of $\mathfrak{v}$ and $\mathfrak{a}$?

Suppose we are interested in the details of vision. Zoom into $\mathfrak{v}$ and break it into $\mathfrak{c}|\mathfrak{s}$, color and shape. The quale at resolution $\mathcal{R}' = \{\mathfrak{c}, \mathfrak{s}, \mathfrak{a}\}$ is

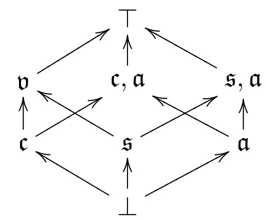

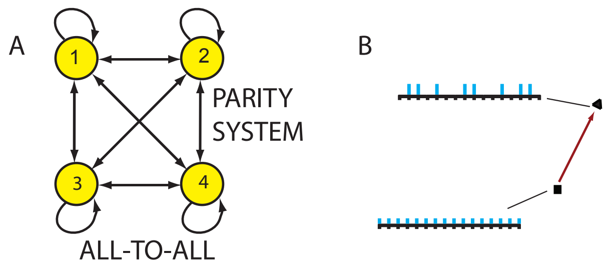

Figure S3: Parity system. The quale generated by a homogeneous parity system consists of a single q-arrow, regardless of the number of elements in the system.

What additional information is available? First, the internal structure of the visual system: q-arrows $\bot \to \mathfrak{v}$ and $\mathfrak{a} \to \top$ are blown up (recall, $\mathfrak{a} = \neg\mathfrak{v}$). Thus using $\mathcal{R}'$ we are able to analyze the relationship between color and shape perception:

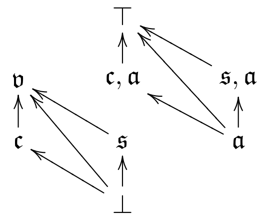

Second, contextual contributions of the visual system. Increasing the resolution introduces two additional transversal q-arrows (starting at $\mathfrak{c}$ and $\mathfrak{s}$), which show how context derived from color perception and shape perception respectively change the information generated by the auditory system:

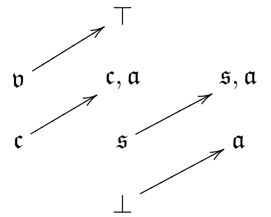

Two resolutions of particular interest are given by partitioning connections according to their *target* or *source* elements. In the first case, q-arrows specify the information generated *by* elements in some context; in the second case, q-arrows specify the information generated *about* elements in some context.

SI-7 Similarity and symmetries

Certain experiences are more alike than others. This suggests that it may be possible to develop measures of similarity – weakening the notion of isomorphism – that can be applied to the different qualia generated by a system or class of systems. Similarity measures are discussed in a different framework in [25, 26].

SI-7.1 The homogeneous parity system

Fig. S3A shows a parity system consisting of 4 binary elements with all-to-all connectivity (including self-connections), where each element fires or is silent according to whether it receives an odd or even number of spikes respectively. In [5] we showed that a parity system generates $\phi = 1$ bit of integrated information regardless of the number n of elements in the system. Panel B shows the shape of the quale generated by the parity system. The abstract lattice $\mathcal{L}(X)$, which contains a vast number of points as n becomes large, collapses into a single q-arrow with effective information of 1 bit. A set of connections $\mathfrak{a}$ maps onto $\top(x_1)$ if and only if it contains (at least) all connections targeting an element. Any smaller set of connections generates no information at all, due to the extreme brittleness of the parity mechanism. Any larger set of connections is redundant.

The quale of an n element parity system looks the same as that of a two element *COPY* system, and a 3 element

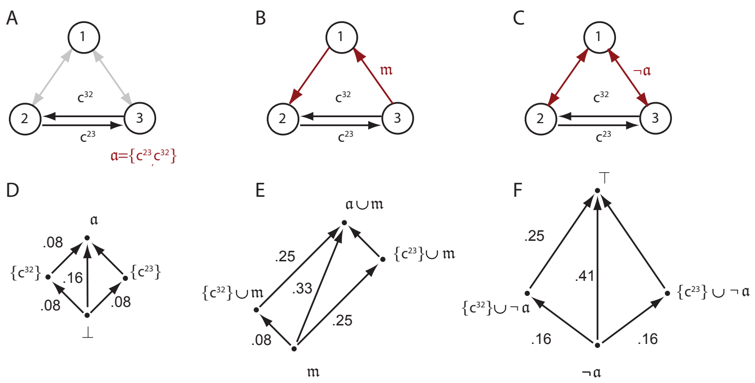

Figure S4: Context in a system of three silent AND-gates. The quale generated by a triple of *AND*-gates consists of 51 distinct points (collapsing from 64 in the abstract lattice). Rather than draw the entire quale, we consider subshapes generated by connections in $\mathfrak{a} = \{c^{23}, c^{32}\}$ in three different context, panels ABC: : null, $\mathfrak{m} = \{c^{31}, c^{12}\}$ and the full context $\neg\mathfrak{a}$. (DEF): the shapes generated by connections in $\mathfrak{a} = \{c^{23}, c^{32}\}$ in the respective contexts. In the null context, the shape generated by $\mathfrak{a}$ is determined by the mechanisms of elements n^2 and n^3 alone. The same connections in the full context generate a different, much larger, subshape that depends on the architecture (mechanisms and connectivity) of the entire system and is not reducible to the mechanisms of the two elements.

system containing a single *XOR*-gate. The qualia generated by the different systems are not isomorphic, since they contain different numbers of elements, and so the qualia spaces cannot be mapped to one another by relabeling elements. Nevertheless, we expect the minimal experiences generated by the systems to be similar. The quale of the parity system is both highly symmetric (a single q-arrow is invariant under many rotations or relabelings) and highly redundant (since the abstract lattice collapses onto two points). Symmetries in a more interesting example, the *AND*-triple, are analyzed in some detail below. In general, the more complex the system, the more symmetries should break; it is extremely unlikely that two biological systems are capable of generated identical experiences.

SI-7.2 The silent AND-triple

Fig S4 shows q-arrows generated by a triple of silent *AND*-gates. The contribution of connections $\mathfrak{a} = \{c^{23}, c^{32}\}$ is shown in three different contexts: the null context $\perp$, $\mathfrak{m} = \{c^{31}, c^{12}\}$, and the full context $\neg\mathfrak{a}$. Adding the connections to the bottom of the quale, panel A, produces a group of q-arrows that generate very little effective information, $ei\big(X_0(\mathfrak{a}, x_1)\big) = .16$ bits. When the context in which the connections are engaged is increased, as in panels BC, the effective information generated by the q-arrows increases to .33 and .41 bits, and the shape of the group changes. The shape taken in isolation, as in panel A, is completely generic: the interactions could have occurred in any network at all. As context is added, the shape becomes increasingly dependent on other mechanisms in the network.

The *AND*-triple contains 6 connections, so the abstract lattice $\mathcal{L}(X)$ contains $2^6 = 64$ subsets. Not all of these subsets generate distinct repertoires; some of the repertoires collapse into each other due to symmetries in the network architecture so that the quale contains 51 points instead of 64. The thirteen symmetries that cause the quale to collapse from 64 potential points to 51 actual points are shown in panel Fig. S5. These are connections that specify identical repertoires, or alternatively, across which redundant interactions occur. The more redundancies there are in the system's mechanism, the less rich the quale.

Analyzing the symmetries in the system, as in Fig. S5 provides an additional perspective on the quale. A simple quale will have many symmetries, and conversely a complex quale will have few.

SI-7.3 The moduli space of qualia

If we focus attention on the qualia generated by a single system, it is natural to consider the moduli space of all qualia that can possibly be generated by that system. A moduli space is a space that parametrizes other spaces. Thus, points of the moduli space of qualia parametrize distinct qualia generated by the system as it

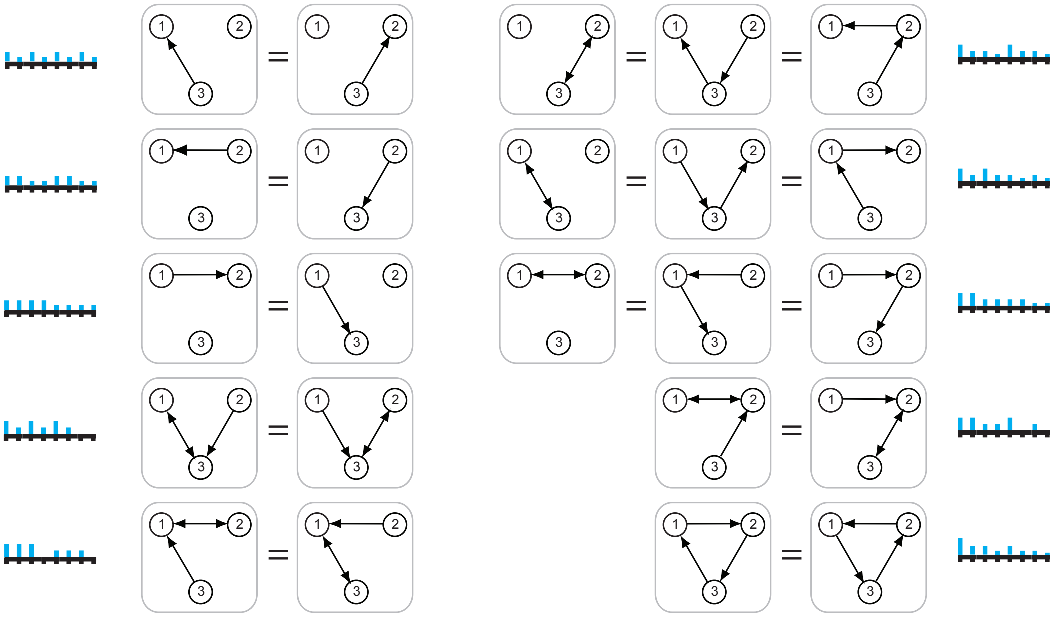

Figure S5: Symmetries in the AND-triple. A silent tripe of *AND*-gates generates a quale with 51 points, collapsing together 13 points in the abstract lattice $\mathcal{L}(X)$. The 13 symmetries causing the collapse are shown, along with the actual repertoires specified.

ranges over possible outputs[2]. It may be possible to construct distance-like measures on the moduli space. As a possible example, consider the color pyramid. Extensive psychophysical experiments [28] have converged on a 3-dimensional representation of colors in a pyramidal color space, with dimensions for hue, saturation and lightness. The color pyramid represents similarities *between* experiences and therefore might emerge as a property of the moduli space of qualia, which parametrizes different experiences. The color pyramid is discovered by probing the system with different stimuli and investigating behavioral responses, which can be thought of as applying a behaviorally determined similarity measure to (a piece of) the moduli space of qualia.

SI-7.4 Measuring similarity

It should be possible to measure similarity of experiences both within a single system system and across systems. For example, the fact that the shape of the quale generated by the parity system is indifferent to the number of elements may have implications for consciousness. Severe epilepsy patients who have had their corpus callosum cut appear to possess two distinct consciousness, one localized in each hemisphere. Particularly surprising is that the left hemisphere reports similar experiences to an intact brain. It is possible that the quale generated by a single hemisphere is similar, in a quantifiable sense, to the quale generated by the entire brain.

Constructing similarity measures can be approached in two ways. First, from a behavioral perspective: identify two q-arrows within the system as similar if future system activity is similar *ceteris paribus*. This requires a choice of appropriate behavioral measures. For example, “does a particular element fire at $t = 2$?” could be taken as the equivalent of a yes/no question. Alternatively, and more fundamentally, similarity can be interpreted as a geometric problem: given two lattices embedded in two qualia spaces, how can we measure the difference between them? This perspective opens the door to a wealth of mathematical machinery that deals with similarity and symmetry.

SI-8 Notation

The notation used in [5] has been modified in this paper as follows.

[2]The qualia space introduced in [27] is more closely related to the moduli space of qualia than to the quale defined in the main text.

	Old	New
mechanism	$mech$	$mech = \top = \mathfrak{Conn}_X$
potential repertoire	$p^{max}(X_0)$	$X_0(maxH)$
actual repertoire specified by whole	$p(X_0 \to x_1)$	$X_0(\top, x_1)$
actual repertoire specified by part M^k	$p(M_0^k \to \mu_1^k)$	$M_0^k(\mathfrak{m}^k, \mu_1^k)$, where $\mathfrak{m}^k$ = connections within M^k
actual repertoire specified by $\mathfrak{m} \subset \mathfrak{Conn}_X$		$X_0(\mathfrak{m}, x_1)$
effective information generated by whole	$ei(X_0 \to x_1)$	$ei\Big(X_0(\top, x_1)\Big)$
effective information across a partition	$ei(X_0 \to x_1/\mathcal{P})$	$ei\Big(X_0(\mathfrak{p}, x_1) \to X_0(\top, x_1)\Big)$
state space		$\mathcal{S}(X)$
lattice of submechanisms		$\mathcal{L}(X)$
qualia space		$\mathcal{Q}(X)$
informational relationship (q-arrow)		$X_0(\mathfrak{m}, x_1) \to X_0(\mathfrak{m} \cup \mathfrak{r}, x_1)$
quale generated by a complex in state x_1		$Q(x_1) = Q(mech, x_1)$

SI-9 Explicit description of mechanisms

We precisely describe the mechanisms of elements in the main text:

Gate	Fire iff receives
COPY	1 spike
AND	≥ 2 spikes
OR	≥ 1 spike
XOR	1 spike exactly
PARITY	an ODD number of spikes
BARS	one of 1100, 0110, 0011
MINORITY	≤ 1 spike

The probabilistic NOISY COPY gate fires with $p = \frac{1}{2}$ if it receives a spike and is silent otherwise. The probabilistic PURE NOISE gate fires with $p = \frac{1}{2}$ regardless of whether or not if receives a spike.

References

1. Cover T, Thomas J (2006) Elements of information theory. John Wiley & Sons.
2. Jaynes E (1985) Entropy and Search Theory. In: Smith C, Grandy W, editors, Maximum-entropy and Bayesian Methods in Inverse Problems. Springer.
3. Jaynes E (1957) Information theory and statistical mechanics. Phys Rev 106:620–630.
4. Pearl J (2000) Causality: models, reasoning and inference. Cambridge University Press.
5. Balduzzi D, Tononi G (2008) Integrated Information in Discrete Dynamical Systems: Motivation and Theoretical Framework. PLoS Comput Biol 4:e1000091. doi:10.1371/journal.pcbi.1000091.
6. Bateson G (1979) Mind and nature: A necessary unity. New York: Dutton.
7. Rovelli C (1996) Relational quantum mechanics. International Journal of Theoretical Physics 35:1637–1678.

8. Smerlak M, Rovelli C (2007) Relational EPR. Foundations of Physics 37:427–445.
9. Reed M, Simon B (1980) Functional analysis. Academic Press.
10. Rudin W (1987) Real and complex analysis. McGraw-Hill.
11. Johnstone P (1986) Stone spaces. Cambridge University Press.
12. Marr D (1982) Vision: A Computational Investigation Into the Human Representation and Processing of Visual Information. W.H. Freeman.
13. Davey B, Priestley H (2002) Introduction to Lattices and Order. Cambridge University Press.
14. Stanley R (2000) Enumerative Combinatorics: Volume I. Cambridge University Press.
15. Jordan M (2004) Graphical models. Statistical Science 19:140–155.
16. Amari S (2001) Information geometry on hierarchy of probability distributions. IEEE Trans Inf Theory 47:1701–1711.
17. Nakahara H, Amari S (2002) Information-geometric measure for neural spikes. Neural Computation 14:2269–2316.
18. Ay N, Olbrich E, Bertschinger N, Jost J (2006) A unifying framework for complexity measures of finite systems. In: Proceedings of ECCS06, European Complex Systems Society, Oxford, UK. pp. ECCS06–174.
19. Tononi G, Sporns O, Edelman G (1994) A measure for brain complexity: Relating functional segregation and integration in the nervous system. Proc Nat Acad Sci 91:5033–5037.
20. Amari S, Nagaoka H (2000) Methods of Information Geometry, volume 191 of *AMS Translations of Mathematical Monographs*. Oxford University Press.
21. Vedral V, Plenio M, Rippin M, Knight P (1997) Quantifying Entanglement. Phys Rev Lett 78:2275–2279.
22. MacLane S (1998) Categories for the Working Mathematician. Springer.
23. Garcia-Carpintero M, Macia J, editors (2006) Two-dimensional semantics. Oxford University Press.
24. Feldman J (2003) A catalog of Boolean concepts. J Math Psychol 47:75–89.
25. Churchland P (1998) Conceptual similarity across sensory and neural diversity: The Fodor/Lepore challenge answered. J Philosophy XCV.
26. Laakso A, Cottrell G (2000) Content and cluster analysis: assessing representational similarity in neural systems. Phil Psych 13:47–76.
27. Stanley R (1999) Qualia space. J Consc Studies 6:49–60.
28. Palmer SE (1999) Color, consciousness, and the isomorphism constraint. Behav Brain Sci 22:923–943.
